# Effects of coffee roasting technologies on cup quality and bioactive compounds of specialty coffee beans

**DOI:** 10.1002/fsn3.1904

**Published:** 2020-10-08

**Authors:** Muluken Bolka, Shimelis Emire

**Affiliations:** ^1^ Institute of Technology Hawassa University Hawassa Ethiopia; ^2^ Addis Ababa Institute of Technology Addis Ababa University Addis Ababa Ethiopia

**Keywords:** acrylamide, caffeine, chlorogenic acids, coffee roaster, high‐quality coffee, trigonelline

## Abstract

The effects of drum, fluidized bed, and traditional type of coffee roasting technologies on the cup quality and bioactive compounds of Yirgacheffe, Harar, and Sidama variety specialty coffee beans grown in Ethiopia were investigated at light, medium, and dark degree of roast from 150°C to 200°C for 7 to 15 min. No significant differences in cup quality were detected among the roasted coffee varieties disregard of the type of roasters. Varietal difference was found to have significant (*p* < .05) effect on caffeine content of the coffee beans. A significant reduction in trigonelline and total chlorogenic acids content of the coffee beans was observed during roasting process, with darker roasts attaining the least values. Drum roaster was found to be the best type of coffee roaster for specialty coffee beans at medium degree of roast with the highest cup quality, optimum bioactive compounds content, and minimum acrylamide formation. However, traditional roaster resulted at the least average cup score of 80% among the three coffee samples and the highest acrylamide content of 2.306 mg/L for Yirgacheffe coffee sample at light degree of roast. There are still some bottlenecks that need to be addressed via advancements using novel food processing technologies in order to devise the next generation of coffee processing.

## Graphical abstract

The practical aspects can contribute to process high quality and safe coffee products using drum roaster. Thus, the drum roaster technology can open senses of opportunity to manufacture diversified commercial coffee products for Local and International market through larger scale operation and appropriate packaging technology in order to share the global market.

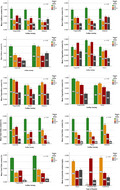

## 1 INTRODUCTION

Coffee is the most worldwide accepted beverage, which has become the second most valuable commodity after oil (Bae et al., 2014). It is the paramount commercialized product with over 3 billion cups of coffee drinks consumed worldwide every day (International Coffee Organization, 2020; Al‐Dalain et al., 2020).

Coffee is a complex mixture of thousands of chemical compounds responsible of its flavor and aroma, carbohydrates, lipids, nitrogenous compounds, vitamins, minerals, alkaloids, and phenolic compounds (Gonzalez & Ramirez‐Mares, 2014). Coffee is rich in many bioactive substances (trigonelline, chlorogenic acid, diterpenes, caffeine), and its consumption has been associated with many beneficial effects (Chu et al., 2009). These include, but not limited to, reduced risk of hepatocellular carcinoma (Johnson et al., 2011), anti‐proliferative effect against some human cancer cell lines (Tai et al., 2010), and therapeutic potential against Alzheimer's disease (Arendash & Cao, 2010), and coffee consumption prevents diabetes and fights liver disease (Cano‐Marquina et al., 2013).

Specialty coffee is coffee which is standardized by the whole coffee process cycle from choosing coffee plantation criteria's till coffee brew serving to client. Specialty coffee flavor is focused on fruity, floral, and acidic notes. The presence of at least five different flavor notes within balance in coffee is valued with higher sensory score (Dejene, 2011; Carvalho et al., 2016; Kelly, 2018). The sensory analysis system for specialty coffee is cup tasting or cupping, according to the Specialty Coffee Association of America's guidelines and cupping protocol (Specialty Coffee Association of America, 2015). In practical terms, this means that the coffee must be able to pass aspect or preliminary grading and cupping tests (Poltronieri & Rossi, 2016).

The coffee roasting process is one of the utmost momentous parts of coffee aroma formation and also has a wide‐ranging influence on the biologically active compounds composition in coffee. It is essential to comprehend the point of the technological parameters of roasting process when the pleasant specialty coffee aroma compounds and health‐beneficial bioactive compounds are at the best ratio (Laukaleja & Kruma, 2019). The degrees of roasting are controlled by roasting time and temperature and are necessary for the required chemical reactions without burning the beans and compromising the flavor of the beverage (Mendes, 2001). The degrees of roasting were qualitatively assessed for color and classified as a light, medium, or dark roast (Bauer et al., 2018; Carvalho et al., 2016). Achieving an ideal roast is a goal that is complicated since coffee beans behave differently and produce distinct results in physical properties, chemical composition, and biological activities when roasted at different conditions (Baggenstoss et al., 2008; Rosa et al., 2016).

Nowadays, there are many types of coffee roasting technologies. Taking into account roasting techniques, coffee beans are conventionally roasted in batch or in continuous systems. The heat can be transferred to the beans by conduction at direct contact with hot metal surfaces, by free or forced convection due to a streaming media (hot air), or by radiation (Wang & Lim, 2014). The most common type of coffee roaster available for home and industrial use is drum roaster. It is a rotating horizontal cylinder that roasts coffee beans placed inside it by continually rotating and heating them by hot air pumped through either the center of the cylinder or through its perforated sides to ensure an even roast. One of the recent developments in coffee roasting technology is fluidized bed roasting (Diallo, 2019) in which high velocity hot gas directed toward the beans, usually from the bottom of the roasting machine, so that the gases heat and move the floating beans simultaneously.

By knowing the type of coffee roaster used and the desired degree of roasting (methods of roasting, time and temperature profiles, and coffee's load) influences on coffees aroma, color, and bioactive compound composition, which in turn contributes to maintain high coffee cupping score without losing the valuable bioactive compounds (Laukaleja & Kruma, 2019). Therefore, the aim of this research was to investigate the changes in the bioactive compounds (caffeine, trigonelline, and total chlorogenic acids) of roasted specialty coffee varieties and compare their cup quality at different degree of roast using the three types of coffee roasting technologies. The concentration of acrylamide formed in Yirgacheffe coffee roasted at different degree of roast was also investigated due to its carcinogenic risks.

## 2 MATERIALS AND METHODS

### 2.1 Raw material preparation

Three Ethiopian specialty green coffee beans (coffee Arabica species) were collected from Yirgacheffe, Harar, and Sidama coffee growing areas due to their unique sensory characteristics and high global market value. All the three coffee varieties have had similar annual temperature (15–35°C), annual rainfall (1000–2000 mm), harvesting season (September to January), and growing practices (organic, tree shed‐garden). As a result, they have had similar maturation period of 6–9 months (Brando, 2004) and were freshly harvested at fully grown stage during the 2018/19 crop year. Furthermore, all the samples were chosen to be unwashed or dry processed for wholesome coffee quality. The raw sample coffee beans varieties were physically examined for defect, odor quality, moisture content, size, and shape. Each sample weighs about 1 kg. The collected sample of raw coffee beans was packed in food grade polyethylene bags and stored at 20°C and 70% RH in air‐conditioned coffee storage facility found in the Ethiopian Coffee and Tea Authority Coffee Quality Inspection and Certification Center (ECTACQICC) in Addis Ababa, Ethiopia.

Each coffee sample was roasted at light, medium, and dark degree of roast using drum roaster (BRZ 4, Probat^®^, 2009), fluidized bed roaster (V3, IKAWA^®^ Pro, 2018), and traditional oven top roaster. The roasting temperature was set to a maximum of 200°C, and the roasting time was limited to 15 min to ensure a dark roast at the end of each roasting process (Górecki & Hallmann, 2020). Then, the roasted coffee beans were air cooled to halt exothermic reaction and allowed to stay overnight in order to allow sufficient time for full flavor development and CO_2_ degassing. The roasted coffee beans were grinded at medium grind size of about 850 µm using electronic laboratory scale coffee grinder (VTA6S, MAHLKÖNIG GmbH & Co., 2009). Afterward, the ground coffee samples were separately labeled, packed in airtight odor‐free food grade polyethylene bags, and stored at 4°C and 70% RH until analyses were performed.

For cupping evaluation, the coffee brew was prepared from each sample of roasted and ground coffee with 1:18 (w/w) ratio of coffee powder and water, according to the method described by Sualeh and Mekonnen (2015). For bioactive compounds analyses, sample coffee extracts were prepared by solid–liquid extraction of roasted and ground coffee with distilled water at a ratio of 1:100 (w/w). The coffee–water mixture was boiled at 95°C for 20 min and stirred using magnetic stirrer at 300 rpm (Yellow line magnetic IKA, 2008), cooled, and filtered through 0.45µm Whatman filter paper. For acrylamide quantification, the above extract was further treated with ethanol and Carrez reagents in order to precipitate polysaccharides and proteins, respectively, centrifuged at 15,000 G for 15 min (90–1 Electrical Centrifuge, 2003), concentrated at 55°C using rotary evaporator (IKA RV 10 Digital V, 2016), and purified with a 10 µm pore size polyethylene frit (Sep‐Pak® Classic C18, Waters, Milford, 2018) to eliminate the main interferences (Soares et al., 2006).

### 2.2 Cupping of roasted coffee beans

The cup quality of medium roasted coffee samples was evaluated by three professional cuppers for aromatic intensity, aromatic quality, color, acidity, astringency, bitterness, body, flavor, and overall acceptability according to ECX (2015) standard coffee cupping protocol. The cup score of each sample was independently filled by each panelist on a separate cupping form, and the average of the total scores was taken as a final score.

### 2.3 Color analysis

The color of the roasted coffee samples was analyzed using a handheld Coffee Roast Analyzer (JAV‐RDA‐H, JAVALYTICS^TM^, Madison Instruments Inc., USA, 2012) to ensure proper degree of roast for grinding and making coffee with pleasant sensory quality (Beattie, 2012). The equipment was calibrated against a gray reference tile before each measurement. And the color value was expressed in Agtron color reference scale according to the protocol of Specialty Coffee Association of America (Specialty Coffee Association of America, 2015).

### 2.4 Bioactive compounds quantification

The caffeine, trigonelline, and total chlorogenic acids (5‐CQA) content of the roasted and ground coffee samples were simultaneously determined using a high‐performance liquid chromatography method adopted from Vignoli et al. (2014). The analysis was performed using an HPLC system (Agilent 1,260 Infinity II) with a diode array detector (DAD). Chromatographic separation was made on a normal phase C18 column (4.6 × 100 mm, 5 μm), with the column temperature held at 25°C. The mobile phase consisted of 5% aqueous acetic acid as solvent A and 100% acetonitrile as solvent B. The chromatography was run under isocratic condition by using 4:96 percent acetic acid to acetonitrile solvent ratio at a flow rate of 0.7 ml/min.

### 2.5 Determination of acrylamide concentration

The acrylamide concentration in the roasted coffee samples was determined using UV‐Vis spectrophotometer following a validated analytical method according to Bicchi et al. (1995) and Soares et al. (2006). UV‐Vis Spectrophotometer (Agilent, Cary 300, version 12.00, USA, 2015) instrument was used for the acrylamide analysis. The wavelength range was set to 700–200 nm. A mixture of 1:1 ratio absolute ethanol and distilled water was used to prepare 2 g/L stock solution and working standard solutions of 0.4 mg/L, 1 mg/L, 2 mg/L, and 4 mg/L acrylamide concentration from a standard acrylamide (Sigma Aldrich). The maximum absorbance of the samples was recorded at a wavelength of 220 nm.

### 2.6 Experimental design and statistical data analysis

Three factorial experimental design at three levels of treatments was used for the study. The selected factors were (A) coffee varieties, (B) type of coffee roasters, and (C) degree of roast. Three coffee varieties, namely Yirgacheffe, Harar, and Sidama, were roasted using three type of coffee roasters (drum, fluidized bed, and oven top roaster), at three degree of roasts (light, medium, and dark). The experimental design was completely randomized, with a maximum of three observations. The obtained experimental results or data were analyzed and interpreted by using analysis of variance (ANOVA) at a level of 5% significance using Design Expert software version 6.0.8.

## 3 RESULTS AND DISCUSSION

### 3.1 Color analysis of roasted coffee beans

The color analysis result of roasted coffee beans samples was presented in Table 1. The color value of the roasted sample coffee beans ranged from 97.8 to 25.1, that is, “Extremely Light” to “Very Dark” according to the SCAA Agtron color scale. According to (Paul, 2012), this is well in the range of “Light” to “Dark” when converted to common naming used to describe degree of coffee roast.

**Table 1:** Specialty Coffee Association of America color value of sample coffee beans roasted at different conditions

No.	Sample code	Instrument reading		Common name
		Color value	Color name	
1	01Dl	90.3	Extremely Light	Light
2	01DM	62.7	Medium Light	Medium
3	01DD	56.6	Medium	Medium
4	01Fl	81.8	Very Light	Light
5	01FM	68.2	Medium Light	Medium
6	01FD	33.5	Dark	Dark
7	01Tl	82.9	Very Light	Light
8	01TM	70.1	Light	Medium
9	01TD	46.8	Moderately Dark	Medium Dark
10	02Dl	89.1	Very Light	Light
11	02DM	91.6	Extremely Light	Light
12	02DD	37.7	Dark	Dark
13	02Fl	97.8	Extremely Light	Light
14	02FM	66.1	Medium Light	Medium
15	02FD	38.9	Dark	Dark
16	02Tl	81.7	Very Light	Light
17	02TM	75.3	Light	Medium
18	02TD	25.1	Very Dark	Dark
19	03Dl	90.8	Extremely Light	Light
20	03DM	87.4	Very Light	Light
21	03DD	43.0	Moderately Dark	Dark
22	03Fl	94.6	Extremely Light	Light
23	03FM	65.0	Medium Light	Medium
24	03FD	38.2	Dark	Dark

Note

All the results are expressed as the mean value of three measurements.

Where 1. 01Dl, 01DM, 01DD, 02Dl, 02DM, 02DD, 03Dl, 03DM, and 03DD are drum roasted sample coffees; 2. 01Fl, 01FM, 01FD, 02Fl, 02FM, 02FD, 03Fl, 03FM, and 03FD are fluidized bed roaster coffees; 3. 01Tl, 01TM, 01TD, 02Tl, 02TM, and 02TD are sample coffees roasted by traditional roaster.

### 3.2 Moisture content of raw coffee beans and coffee roasted at different conditions

Based on the results shown in the Table 2, the moisture content of the raw sample coffee beans was in the optimum range of 8%–12% (ECX, 2015) and can be stored at room temperature without growing fungus or mold. A small difference among the moisture contents of the three coffee varieties may be due to variations in climate and weather conditions, drying time during processing, storage conditions, and way of handling during transportation.

**Table 2:** Moisture content of raw coffee beans

Green (raw) coffee beans	Moisture content (%) in wet basis
Yirgacheffe	9.8 ± 0.1
Harar	10.0 ± 0.2
Sidama	10.3 ± 0.1

Note

Results are expressed as the average of triplicate samples with mean values ± 0.25 *SD*.

Further, the changes in the moisture content of the coffee beans were noticeable during the roasting process. The moisture contents of Yirgacheffe coffee beans roasted using the three types of roasters are presented in Table 3. According to analysis of variance, the moisture content of the roasted sample coffee beans showed significant (*p* < .05) difference among the three degree of roasts and types of roasting technologies. The results ranged from 1.31% to 3.48%, being higher for lighter roast degrees and lower for darker roast. The least moisture content was obtained at dark roast degree using fluidized bed roaster and for longer roasting times. This is because of a more moisture loss enhanced by hot air circulation in the fluidized bed roaster.

**Table 3:** Moisture content of Yirgacheffe coffee roasted at different conditions

Type of roaster	Moisture content (%) at various degree of roast		
	Light Roast	Medium Roast	Dark Roast
Drum	3.410	3.204	1.339
Fluidized Bed	3.477	1.884	1.311
Traditional oven top roaster	3.414	1.971	1.323

Note

All results are expressed as the average of duplicate samples with mean values.

### 3.3 Effects of coffee roasting technologies on cup quality of the coffee beans

Among all the coffee varieties, Yirgacheffe coffee showed the highest cupping score using fluidized bed roaster. Whereas the highest cupping scores for Harar and Sidama Coffees were obtained by roasting them using drum roaster. Hence, traditional oven top roaster resulted at the least sensory score for all the coffee sample. However, the cup characteristics values of fragrance, flavor, after taste, acidity, body, balance, uniformity, cup cleanliness, sweetness, and overall acceptability showed no significant (*p* > .05) difference for both coffee variety and type of roaster. Despite this, the top scores for fragrance and body were obtained from Sidama coffee roasted using drum roaster. And the top scores for flavor, after taste, and balance were recorded from Yirgacheffe coffee roasted using fluidized bed roaster as shown in Table 4.

**Table 4:** Cupping scores of roasted coffee beans

Sample code	Cup characteristics										Total Score (100%)
	Fragrance	Flavor	After Taste	Acidity	Body	Uniformity	Balance	Clean cup	Sweetness	Overall acceptability	
01DM	8.00	7.67	7.08	7.08	7.42	10	8.08	10	10	7.83	83.17
01FM	7.83	8.08	8.07	7.83	7.83	10	8.25	10	10	8.58	86.42
01TM	7.01	7.07	7.51	7.42	7.67	10	7.92	10	10	8.04	82.5
02DM	7.58	7.42	7.33	7.25	7.33	10	7.75	10	10	7.67	82.33
02FM	7.05	7.17	6.92	7.25	7.75	10	7.67	10	10	7.51	81.25
02TM	7.42	7.08	7.08	6.58	7.03	10	7.5	10	10	7.33	80.06
03DM	8.25	7.58	7.58	7.67	8.08	10	8.08	10	10	8.04	85.25
03FM	7.92	7.42	7.58	7.75	7.83	10	8.08	10	10	7.83	84.42

Note

All values are average cupping score of three professional cuppers.

Where: 01DM: Yirgacheffe coffee medium roasted by drum roaster; 02DM: Harar coffee medium roasted by drum roaster; 03DM: Sidama coffee medium roasted by drum roaster; 01FM: Yirgacheffe coffee medium roasted by fluidized bed roaster; 02FM: Harar coffee medium roasted by fluidized bed roaster; 03FM: Sidama coffee medium roasted by fluidized bed roaster; 01TM: Yirgacheffe coffee medium roasted by traditional roaster; 02TM: Harar coffee medium roasted by traditional roaster.

### 3.4 Effects of coffee roasting technologies on composition of bioactive compounds

**Figure 1:**
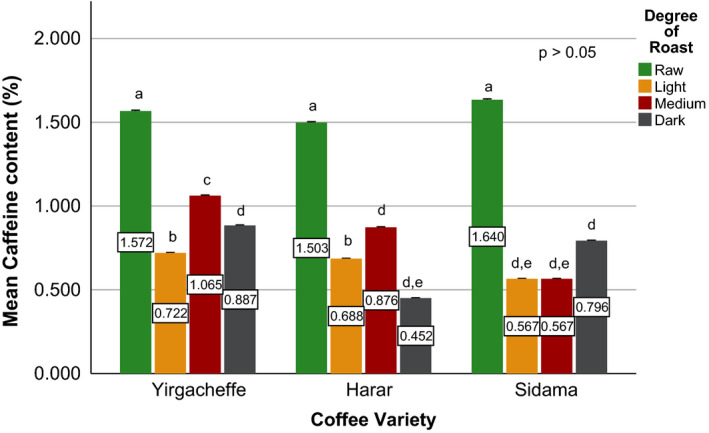
Caffeine content of sample coffee beans roasted by drum roaster. Different letters over bars in the same analysis mean that samples are significantly (*p* < .05) different, and same letters indicate that samples are not significantly (*p* > .05) different

**Figure 2:**
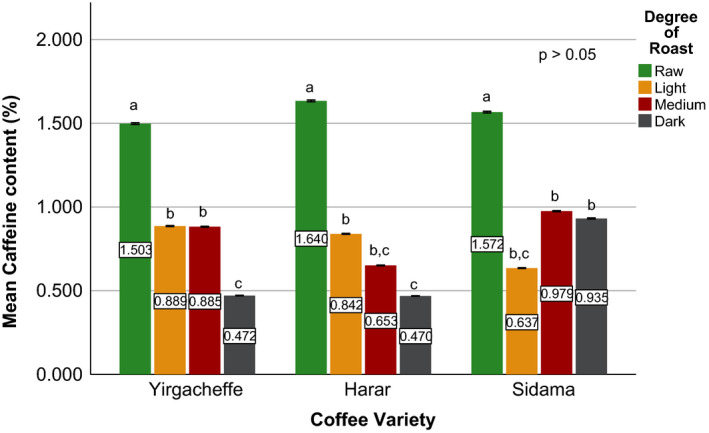
Caffeine content of sample coffee beans roasted by fluidized bed roaster. Different letters over bars in the same analysis mean that samples are significantly (*p* < .05) different, and same letters indicate that samples are not significantly (*p* > .05) different

**Figure 3:**
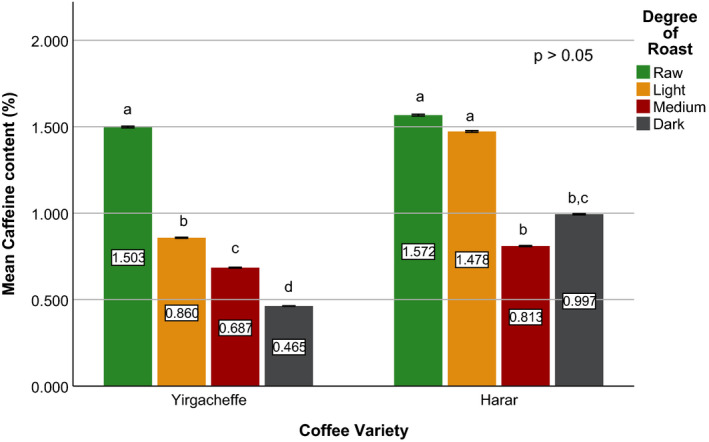
Caffeine content of sample coffee beans roasted by traditional roaster. Different letters over bars in the same analysis mean that samples are significantly (*p* < .05) different, and same letters indicate that samples are not significantly (*p* > .05) different

**Figure 4:**
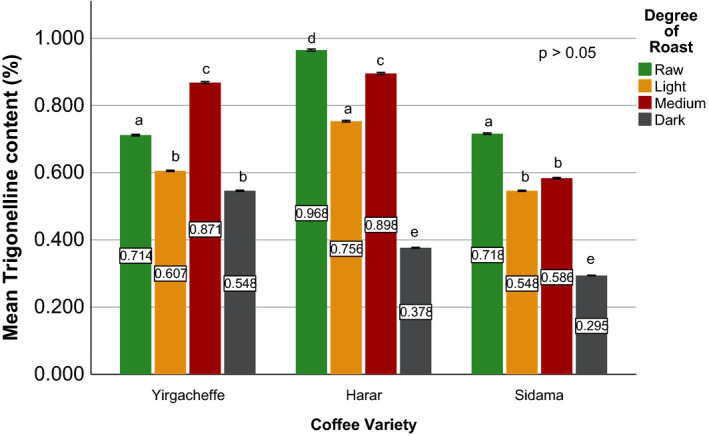
Trigonelline content of sample coffee beans roasted by drum roaster. Different letters over bars in the same analysis mean that samples are significantly (*p* < .05) different, and same letters indicate that samples are not significantly (*p* > .05) different

**Figure 5:**
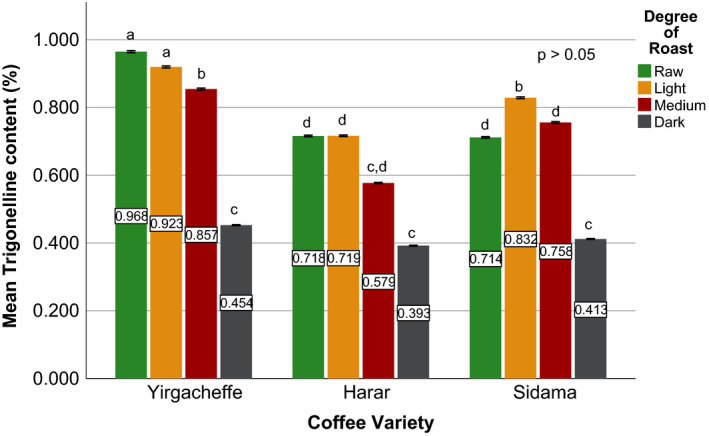
Trigonelline content of sample coffee beans roasted by fluidized bed roaster. Different letters over bars in the same analysis mean that samples are significantly (*p* < .05) different, and same letters indicate that samples are not significantly (*p* > .05) different

**Figure 6:**
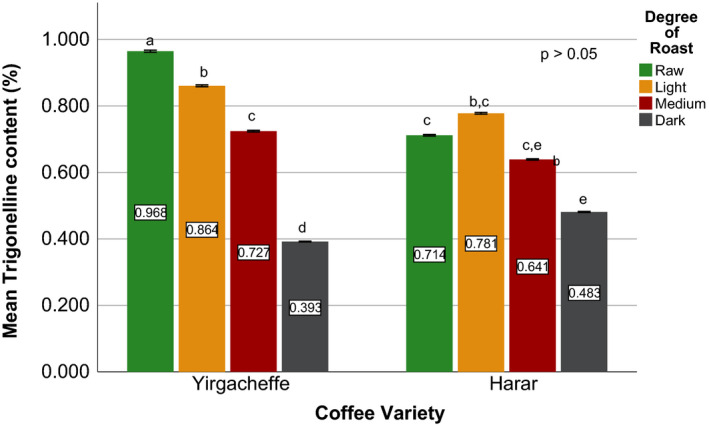
Trigonelline content of sample coffee beans roasted by traditional roaster. Different letters over bars in the same analysis mean that samples are significantly (*p* < .05) different, and same letters indicate that samples are not significantly (*p* > .05) different

**Figure 7:**
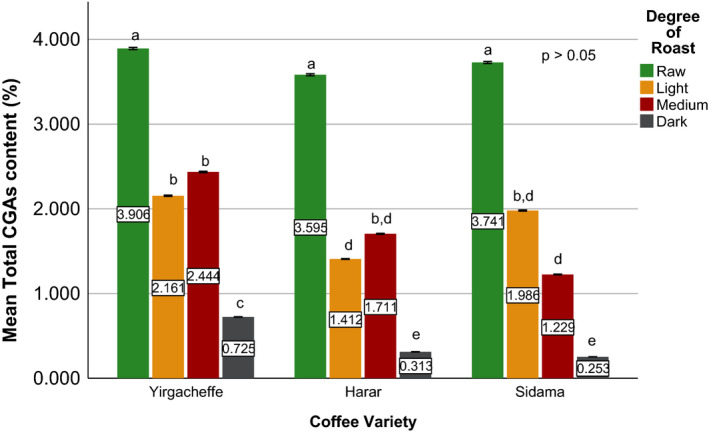
Total chlorogenic acids content of sample coffee beans roasted by drum roaster. Different letters over bars in the same analysis mean that samples are significantly (*p* < .05) different, and same letters indicate that samples are not significantly (*p* > .05) different

**Figure 8:**
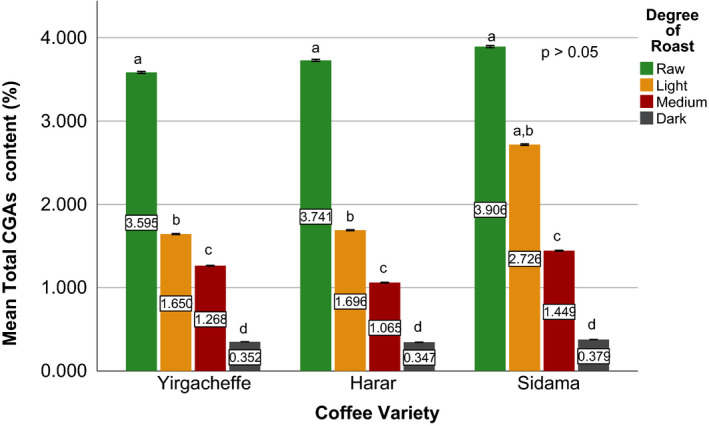
Total chlorogenic acids content of sample coffee beans roasted by fluidized bed roaster. Different letters over bars in the same analysis mean that samples are significantly (*p* < .05) different, and same letters indicate that samples are not significantly (*p* > .05) different

**Figure 9:**
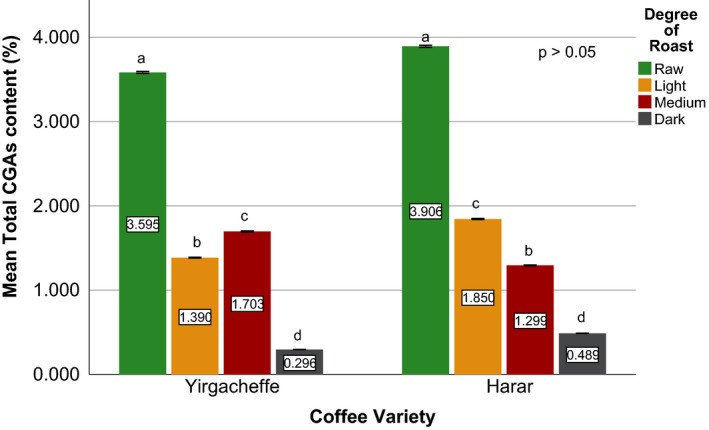
Total chlorogenic acids content of sample coffee beans roasted by traditional roaster. Different letters over bars in the same analysis mean that samples are significantly (*p* < .05) different, and same letters indicate that samples are not significantly (*p* > .05) different

Effects of coffee roasting technologies on composition of bioactive compounds were presented in Figures 1, 2, 3, 4, 5, 6, 7, 8, 9. Caffeine content (Figures 1, 2, 3) of roasted coffee beans, trigonelline content (Figures 4, 5, 6) of coffee beans varieties roasted by means of various roasters and total CGAs content (Figures 7, 8, 9) of coffee samples roasted by various roasters were presented consequently.

According to the research report by Sherge (2017), only few published information is available on the caffeine content of the various coffee varieties grown in Ethiopia. The level of caffeine in all the three specialty coffee beans was above 0.8%, which is the typical caffeine content of most Arabica coffees (Brando, 2004) and the Ethiopian standard for roasted coffee beans and roasted ground coffee (ESA, 2002). This result is in agreement with the works of Farah et al. (2006) and Franca et al. (2005), who compared caffeine levels for samples of different sensory quality coffee beans and found that the highest caffeine levels corresponded to the highest (specialty) quality coffee samples. The caffeine level of Sidama variety was recorded at 1.634%, whereas Yirgacheffe and Harar coffee varieties were measured to have caffeine level of 1.567% and 1.498% (dry weight), respectively. This difference in the caffeine content of the green coffee beans was mainly due to the difference in their variety and growing location (Alonso‐Salces et al., 2009).

A significant reduction in caffeine content of the sample coffee beans was observed during the roasting process (Eggers & Pietsch, 2001; Król et al.,^2019^; Górecki & Hallmann, 2020). Sidama coffee incurred up to 60% reduction in its caffeine level while Harar and Yirgacheffe coffees experienced an average of 53% and 40% loss; respectively. This is due to the increase in the solubility of caffeine in water as the roasting temperature increases. In addition, the caffeine loss was attributed to a drag by bounded water vapor released from the coffee matrix during roasting (Baggenstoss et al., 2008). The apparent higher caffeine percentages at darker degree of roast (i.e., medium or dark roast) in some of the roasted coffees shown in Figures 1, 2, 3 might be due to the decrease in moisture contents as roasting time increases (Vignoli et al., 2014). However, a research conducted by Kitzberger et al. (2014) on seven Arabica coffee cultivars grown under the same climatic conditions shows caffeine stability during roasting. Therefore, more coffee varieties need to be studied to clearly state the effect of roasting on the level of caffeine.

The sensory importance of trigonelline is due to the fact that it undergoes extensive thermal degradation of 50% to 90% (Kalaska et al., 2014) and generating pyridines and pyrroles that make up approximately half of the volatile substances released during coffee roasting process (Rodrigues & Bragagnolo, 2013). Therefore, the lower the trigonelline content in raw coffee beans, the lower the quality of the coffee beverage (Farah et al., 2006).

Quantitative data on the content of trigonelline in Ethiopian coffee varieties are scarce (Sherge, 2017). Among the three raw coffee samples, Harar coffee recorded the highest trigonelline content of 0.965%, followed by Sidama and Yirgacheffe coffees with nearly equal trigonelline content of 0.716% and 0.712%, respectively. A significant reduction in trigonelline content of the coffee beans was observed during the roasting process, with darker roasts attaining the least values. During the early stage inside the drum roaster, Harar, Sidama, and Yirgacheffe coffee showed 22%, 24%, and 15% reduction in their trigonelline content, respectively. However, as the roasting processes progresses from light to medium degree of roast, a sharp increase in the trigonelline content was observed. The highest trigonelline level was recorded at medium degree of roast for all the three coffee varieties, with Harar obtaining 0.895% w/w followed by Yirgacheffe and Sidama coffees attaining 0.868% and 0.584% w/w; respectively. This may explain why medium roasted coffees are usually preferred by professional coffee cuppers or Q‐Graders as the best degree of roast for sensory or cupping test.

As seen on Figure 5, fluidized bed roaster brought about less negative impact on the trigonelline content of all the coffee samples during the early stage of roasting process. At light degree of roast, Harar Coffee showed only 5% reduction from its initial trigonelline content, whereas Yirgacheffe coffee showed 16% rise in its trigonelline content while Sidama coffee showed nearly no change. However, a steady decrease in trigonelline content was observed at medium degree of roast. Therefore, the highest trigonelline level of all the three coffee varieties was recorded at light degree of roast, with Harar obtaining 0.919% w/w while Yirgacheffee and Sidama coffee attaining 0.829% and 0.717% w/w, respectively. As a result, light degree of roast may be recommended for professional coffee roasters who wish to obtain the best cup qualities of their coffee using fluidized bed roasters.

At light degree of roast, traditional oven top roaster also brought about less degradation effect on the initial trigonelline content of the two coffee samples. Yirgacheffe coffee obtained a 9% rise while Harar coffee showed nearly 11% reduction in their initial trigonelline content. However, a continuous decrease in their trigonelline content was observed after the beans pass light degree of roast and progress to darker roast. Thus, the maximum trigonelline content was achieved at light degree of roasted where Harar coffee recorded 0.860% w/w and Yirgacheffee attained 0.778% w/w.

According to the obtained results, medium roasted Harar coffee obtained the highest trigonelline content of 0.895% w/w using drum roaster, followed by 0.855% using fluidized bed roaster, and 0.724% using traditional oven top roaster. Similarly, medium roasted Yirgacheffee coffee showed the highest trigonelline content of 0.868% w/w using drum roaster, followed by 0.756% w/w using fluidized bed roaster 0.639%. Medium roasted Sidama coffee also showed the highest trigonelline content of 0.584% w/w using drum roaster and 0.577% using fluidized bed roaster. This indicates that drum roaster maintains better trigonelline level in the coffees than both fluidized bed roaster and traditional roaster. Therefore, drum type of roasters may be the best choices for roasting specialty coffee beans without jeopardizing their distinctive flavor and aroma.

There is only a limited information in order to compare the chlorogenic acids contents of the collected coffee samples with the chlorogenic acids present in other Ethiopian coffee beans (Alonso‐Salces et al., 2009; Moon et al., 2013). An article by Farah et al. (2006) reported that raw Harar coffee beans contain about 5.6% (w/w) of total chlorogenic acids. (Moon et al., 2013), while studying the role of coffee roasting conditions on the level of chlorogenic acids, reported total chlorogenic acid contents of 6.91%, 3.21%, and 1.97% (w/w) in the green, light roasted, and medium roasted coffee beans of Ethiopian origin, respectively. After studying the chemical composition of green coffee beans grown in different parts of Ethiopia, Sherge (2017) reported that the total chlorogenic acids produced during medium roasting of Harar as 1.93% (w/w).

Among the three raw coffee samples, Yirgacheffe coffee obtained the highest total chlorogenic acids content of 3.89% followed by 3.73% for Sidama coffee, and 3.58% for Harar coffee. During the roasting of the sample coffee beans using drum roaster, a significant reduction was observed in their chlorogenic acids level. At light degree of roast, Harar coffee showed 61% reduction followed by Sidama and Yirgacheffe with relatively close 47% and 46% reduction in their chlorogenic acids content, respectively. As the roasting progresses from light to medium degree of roast, Harar and Yirgacheffe coffees showed 21% and 13% increase in their chlorogenic acids level, respectively. However, Sidama coffee incurred 38% decrease from its chlorogenic acids level at light degree of roast. When the beans become dark roasted, they showed tremendous decrease in their original chlorgenic acids content, with 93% loss incurred by Sidama coffee followed by 91% for Harar, and 81% for Yirgacheffe coffee.

As the sample coffee beans progress from raw to dark degree of roast using fluidized bed roaster, a continuous reduction was observed in their chlorogenic acids level. At light degree of roast, both Harar and Sidama coffee showed around 55% reduction in their initial chlorogenic acids content. However, Yirgacheffe showed just 30% reduction from its original chlorogenic acid level. When the sample coffee beans become dark roasted, all of them incurred an average 90% reduction of their original chlorgenic acids level.

When coffee beans roasted using traditional oven top roaster, that is, Harar and Yirgacheffe coffee beans, incurred an average of 90% reduction in their original chlorgenic acids level. However, Yirgacheffe coffee showed better chlorgenic acid level at light degree of roast, whereas Harar coffee obtained higher level of chlorogenic acids at medium degree of roast. The profile and concentration of biologically active compounds in coffee beans mainly depend on the degree of roasting which in turn affects aroma and taste.

### 3.5 Effects of coffee roasting technologies on acrylamide concentration

**Figure 10:**
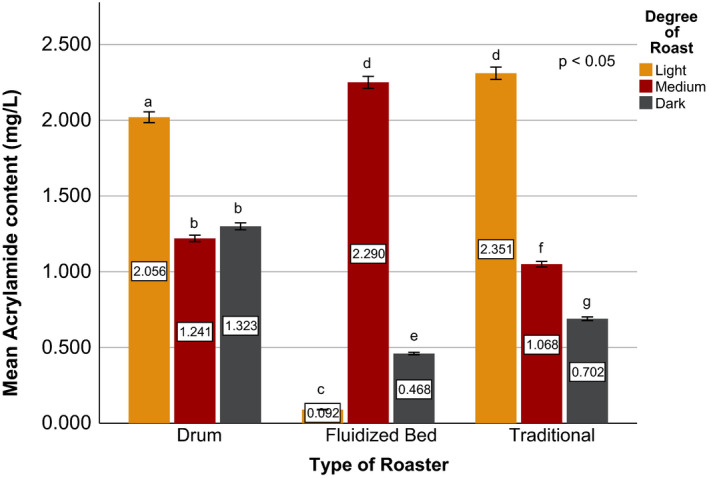
Acrylamide content of sample Yirgacheffe coffee beans roasted by drum, fluidized bed, and traditional type of roasters at light, medium, and dark degree of roast. Different letters over bars in the same analysis mean that samples are significantly (*p* < .05) different

The influence of coffee roasting technologies on acrylamide content of roasted Yirgacheffe coffee was presented in Figure 10. The experiment of coffee roasting technologies on acrylamide concentration showed significant (*p* < .05) differences in the roast results from each type of roaster and degree of roast. The highest acrylamide content of Yirgacheffe coffee was obtained at light degree of roast for both drum and traditional type of roasters. However, fluidized bed roaster resulted at higher acrylamide value in the coffee at medium degree of roast. This could be due to the convective heat transfer involved in fluidized bed roaster producing roasted coffee with lower loss of volatiles than the coffees roasted using the other type of roasters as described by Nagaraju et al. (1997). On the other hand, the lowest content of acrylamide was exhibited at light degree of roast for fluidized bed roaster, at medium degree of roast for drum roaster, and at dark degree of roast for traditional roaster. Among all the types of coffee roasters, fluidized bed roaster resulted at the least acrylamide content in the roasted sample coffee. As a result, fluidized bed roaster might be a better choice to produce low acrylamide roasted coffee and reduce its health risk in humans (Bae et al., 2014).

The correlation coefficients of acrylamide formation with the degree of roast and bioactive compounds content of roasted Yirgacheffe coffee beans are presented in Table 5, 6, 7. The study indicated that acrylamide formation had a highly significant (*p* < .001) positive correlation with degree of roast for coffee beans roasted using drum roaster (Table 5), whereas the results revealed that acrylamide formation had a significant (*p* < .05) correlation with caffeine and no significant (*p* > .05) correlation with trigonelline content of the drum roasted coffee beans (Cuong et al., 2014).

**Table 5:** Correlation coefficients among drum roasted coffees presented in matrix form

	Roast degree	Caffeine	Trigonelline	Total CGAs	Acrylamide
Roast degree	1				
Caffeine	−0.75**	1			
Trigonelline	‐	0.78**	1		
Total CGAs	0.52*	‐	0.75**	1	
Acrylamide	0.96**	−0.90**	−0.43*	‐	1

** Highly significant (*p* < .001).

* Significant (0.01 < *p*<.05), and ‐ None significant (*p* > .05) effect.

**Table 6:** Correlation coefficients among fluidized bed roasted coffees presented in matrix form

	Roast degree	Caffeine	Trigonelline	Total CGAs	Acrylamide
Roast degree	1				
Caffeine	−0.63*	1			
Trigonelline	0.99**	−0.54*	1		
Total CGAs	0.96**	−0.83**	0.92**	1	
Acrylamide	‐	0.72*	‐	‐	1

** Highly significant (*p* < .001).

* Significant (0.01 < *p*<.05), and No significant (*p* > .05) effect.

**Table 7:** Correlation coefficients among traditionally roasted coffees presented

in matrix form	Roast Degree	Caffeine	Trigonelline	Total CGAs	Acrylamide
Roast Degree	1				
Caffeine	0.57*	1			
Trigonelline	0.99**	0.67*	1		
Total CGAs	0.99**	0.62*	0.99**	1	
Acrylamide	0.89**	0.88**	0.94**	0.92**	1

** Highly significant (*p* < .001).

* Significant (0.01 < *p*<.05).

A significant positive correlation was observed between acrylamide formation and caffeine content of coffee beans roasted using fluid bed roaster (Table 6). However, the degree of roast exhibited no significant (*p* > .05) effect on the acrylamide formation. For the traditionally roasted coffee beans, acrylamide formation had a highly significant (*p* < .001) positive correlation with degree of roast and all the bioactive compounds content as presented in Table 7.

## 4 CONCLUSIONS

The results revealed that, Sidama and Harar coffees bring about at the best total cup score using drum roaster, whereas Yirgacheffe coffee achieved the highest score using fluidized bed roaster. Traditional oven top roaster resulted at the least total cup score for all the coffee varieties. Darker roasts resulted at the least amount of trigonelline and total chlorogenic acids content of the coffee beans, and 40%‐60% reduction in caffeine contents of the sample coffees was exhibited throughout the roasting process. Drum roaster maintains better trigonelline level in the coffee beans than fluidized bed roaster and traditional roaster. Drum roasters might be the best choices for roasting specialty coffee beans without jeopardizing their distinctive flavor and aroma.

The results of the present study suggest that acrylamide analysis showed significant differences for type of roaster and degree of roast. The lowest content of acrylamide in the roasted coffee sample was exhibited at light degree of roast using fluidized bed roaster, at medium degree of roast using drum roaster, and at dark degree of roast using traditional roaster. Based on the obtained results, drum roaster is highly desirable to minimizing acrylamide formation at medium degree of roast while maintaining optimum cup quality and bioactive compounds content of the coffee.

Perceiving all the standardized procedures of the whole coffee process cycle of coffee roasting technologies, it is possible to maintain the quality of coffee. In a nutshell, explorations of value addition to coffee via integration of emerging techniques and applications of bioengineering principles in food processing can enhance coffee industry business development specifically in Africa and worldwide. It is very important to acquire enforced roasting standards for commercial coffee roasting and processing industries in Ethiopia in order to maintain high quality and safe coffee products.

## CONFLICT OF INTEREST

The authors declare no financial or commercial conflict of interest. The authors affirm that this manuscript is an honest, accurate, and transparent account of the study being reported.

## AUTHORS’ CONTRIBUTIONS

The first author was responsible for the conceptualization, data curation, formal analysis, investigation, methodology, resources, validation, writing‐original draft, and writing‐review and editing. The second author also contributed to the conceptualization, methodology, writing‐original draft, writing‐review and editing, project administration, and supervision. Both authors contributed in the same way to the preparation of the manuscript and approved the final version for publication.

## STUDIES IN HUMANS AND ANIMALS

This study does not involve any human or animal testing.

## Supporting information

Appendix

## References

[fsn31904-bib-0001] Al‐Dalain, S. Y. , Haddad, M. A. , Parisi, S. , Al‐Tarawneh, M. A. , & Qaralleh, H. (2020). Determination of macroelements, transition elements, and anionic contents of commercial roasted ground coffee available in Jordanian Markets. Beverages, 6(16), 7 10.3390/beverages6010016

[fsn31904-bib-0003] Alonso‐Salces, R. M. , Serra, F. , Reniero, F. , & HÉberger, K Á. (2009). Botanical and geographical characterization of green coffee (*Coffea arabica* and *Coffea canephora*): Chemometric evaluation of phenolic and methylxanthine contents. Journal of Agricultural and Food Chemistry, 57, 4224–4235. 19298065 10.1021/jf8037117

[fsn31904-bib-0004] Arendash, G. W. , & Cao, C. (2010). Caffeine and coffee as therapeutics against Alzheimer’s disease. Journal of Alzheimer Disease, 20(1), 117–126. 10.3233/JAD-2010-091249 20182037

[fsn31904-bib-0005] Bae, J. H. , Park, J. H. , Im, S. S. , & Song, D. K. (2014). Coffee and health: Mini review. Integrative Medicine Research, 3, 189–191. 28664096 10.1016/j.imr.2014.08.002 PMC5481750

[fsn31904-bib-0006] Baggenstoss, J. , Poisson, L. , Kaegi, R. , Perren, R. , & Escher, F. (2008). Coffee roasting and aroma formation: Application of different time temperature conditions. Journal of Agricultural and Food Chemistry, 56(14), 5836–5846. 18572953 10.1021/jf800327j

[fsn31904-bib-0007] Bauer, D. , Abreu, J. , Jordão, N. , Rosa, J. , Freitas‐Silva, O. , & Teodoro, A. (2018). Effect of roasting levels and drying process of *Coffea canephora* on the quality of bioactive compounds and cytotoxicity. International Journal of Molecular Sciences, 19(11), 3407 10.3390/ijms19113407 PMC6274859 30384410

[fsn31904-bib-0008] Beattie, M. (2012). Coffee roasting sample procedures & equipment. Paper presented at the Golden Bean.

[fsn31904-bib-0009] Bicchi, C. P. , Panero, O. M. , Pellegrino, G. M. , & Vanni, A. C. (1995). Characterization of green and roasted coffees through the chlorogenic acid fraction by HPLC‐UV and principal component analysis. Journal of Agricultural and Food Chemistry, 43(6), 1549–1555.

[fsn31904-bib-0010] Brando, C. H. J. (2004). Harvesting and Green Coffee Processing In Nicolas W. J. (Ed.), Coffee: Growing, processing, sustainable production, a guidebook for growers, processors, traders, and researchers (1st ed, pp. 604–714). WILEY‐VCH.

[fsn31904-bib-0011] Cano‐Marquina, A. , Tarín, J. , & Cano, A. (2013). The impact of coffee on health. Maturitas, 75(1), 7–21. 23465359 10.1016/j.maturitas.2013.02.002

[fsn31904-bib-0110] Carvalho, A. M. , Rezende, J. C. , Rezende, T. T. , Ferreira, A. D. , Rezende, R. M. , Guimarães Mendes, A. N. , & Carvalho, G. R. (2016). Relationship between the sensory attributes and the quality of coffee in different environments. African Journal of Agricultural Research, 11(38), 3607–3614.

[fsn31904-bib-0012] Chu, Y.‐F. , Brown, P. H. , Lyle, B. J. , Chen, Y. , Black, R. M. , Williams, C. E. , Lin, Y.‐C. , Hsu, C.‐W. , & Cheng, I. H. (2009). Roasted coffees high in lipophilic antioxidants and chlorogenic acid lactones are more neuroprotective than green coffees. Journal of Agricultural and Food Chemistry, 57(20), 9801–9808. 19772322 10.1021/jf902095z

[fsn31904-bib-0013] Cuong, T. V. , Ling, L. H. , Quan, G. K. , Tiep, T. D. , Nan, X. , Qing, C. X. , & Linh, T. L. (2014). Effect of roasting conditions on several chemical constituents of Vietnam Robusta coffee. Food Technology, 38(2), s43–56.

[fsn31904-bib-0014] de Gonzalez, M. E. , & Ramirez‐Mares, M. V. (2014). Impact of caffeine and coffee on our health. Trends in Endocrinology and Metabolism, 25(10), 498–592. 10.1016/j.tem.2014.07.003 25124982

[fsn31904-bib-0015] Dejene, T. (2011). Quality and value chain analysis of Ethiopian coffee. (MSc thesis), Addis Ababa University.

[fsn31904-bib-0016] Diallo, A. (2019). A Heated Comparison: Ikawa vs Probat [White paper]. Retrieved from https://caravela.coffee/wp-content/uploads/2019/04/A-Heated-Comparison-Ikawa-vs-Probat-FINAL.pdf

[fsn31904-bib-0017] ECX (2015). Ethiopian Commodity Exchange (ECX): ECX coffee contracts, pp. 14 Retrieved from http://www.ecx.com.et/pages/downloads/contracts/Coffee/CoffeeContracts.pdf

[fsn31904-bib-0018] Eggers, R. , & Pietsch, A. (2001). Technology I: roasting In Clarke R. J., & Vitzthum O. G. (Eds.), Coffee recent developments (1st ed, pp. 90–107). Blackwell Science Ltd.

[fsn31904-bib-0019] Ethiopian Standard Agency (2002) Roasted coffee beans and roasted ground coffee ‐ specification (ES 788:2002). pp. 9 Retrieved from http://www.esa.gov.et/documents/147439/158822/2015+catalogue.pdf

[fsn31904-bib-0020] Farah, A. , Monteiro, M. C. , Calado, V. , Franca, A. S. , & Trugo, E. L. C. (2006). Correlation between cup quality and chemical attributes of Brazilian coffee. Food Chemistry, 98(2), 373–380.

[fsn31904-bib-0021] Franca, A. S. , Mendonca, J. C. F. , & Oliveira, S. D. (2005). Composition of green and roasted coffees of different cup qualities. LWT, 38, 709–715.

[fsn31904-bib-0022] Górecki, M. , & Hallmann, E. (2020). The antioxidant content of coffee and its in vitro activity as an effect of its production method and roasting and brewing time. Antioxidants, 9(4), 308 10.3390/antiox9040308 PMC7222172 32290140

[fsn31904-bib-0023] International Coffee Organization (2019). Coffee Development Report 2019. Growing for prosperity: Economic viability as the catalyst for a sustainable coffee sector. London, UK. Retrieved from http://www.ico.org/documents/cy2018-19/ed-2318e-overview-flagship-report.pdf

[fsn31904-bib-0024] Johnson, S. , Koh, W. P. , Wang, R. , Govindarajan, S. , Yu, M. C. , & Yuan, J. M. (2011). Coffee consumption and reduced risk of hepatocellular carcinoma: findings from the Singapore Chinese Health Study. Cancer Causes and Control, 22(3), 503–510. 21258859 10.1007/s10552-010-9725-0 PMC3400401

[fsn31904-bib-0025] Kalaska, B. , Piotrowski, L. , Leszczynska, A. , Michalowski, B. , Kramkowski, K. , Kaminski, T. , Adamus, J. , Marcinek, A. , Gebicki, J. , Mogielnicki, A. , & Buczko, W. (2014). Antithrombotic effects of pyridinium compounds formed from trigonelline upon coffee roasting. Journal of Agriculture and Food Chemistry, 62, 2853–2860. 10.1021/jf5008538 24650005

[fsn31904-bib-0027] Kelly, C. B. D. (2018). The art of coffee roasting: Investigations into sensor development for the application of controlling coffee roasting (Thesis, Doctor of Philosophy, PhD). The University of Waikato Retrieved from https://hdl.handle.net/10289/11614

[fsn31904-bib-0028] Kitzberger, C. , Scholz, M. , & Benassi, M. (2014). Bioactive compounds content in roasted coffee from traditional and modern *Coffea arabica* cultivars grown under the same edapho‐climatic conditions. Food Research International, 61, 61–66. 10.1016/j.foodres.2014.04.031

[fsn31904-bib-0029] Król, K. , Gantner, M. , Tatarak, A. , & Hallmann, E. (2019). The effect of roasting, storage, origin on the bioactive compounds in organic and conventional coffee (*Caffea arabica*). European Food Research and Technology, 2020(246), 33–39. 10.1007/s00217-019-03388-9

[fsn31904-bib-0030] Laukaleja, I. , & Kruma, Z. (2019). Influence of the roasting process on bioactive compounds and aroma profile in specialty coffee: Review Paper presented at the FOODBALT 2019, 13th Baltic Conference on Food Science and Technology, Jelgava, Latvia, 7–12. 10.22616/FoodBalt.2019.002

[fsn31904-bib-0031] Mendes, L. C. (2001). Optimization of the roasting of robusta coffee (*C. canephora* conillon) using acceptability tests and RSM. Journal of Food Quality and Preference, 12, 153–162.

[fsn31904-bib-0032] Moon, J. L. , Sang, E. K. , Jong, H. K. , Sang, W. L. , & Dong, M. Y. (2013). A study of coffee bean characteristics and coffee flavors in relation to roasting. Journal of the Korean Society of Food Science and Nutrition, 42(2), 255–261.

[fsn31904-bib-0033] Nagaraju, V. D. , Murthy, C. T. , Ramalakshmi, K. , & Rao, P. N. S. (1997). Studies on roasting of coffee beans in a spouted bed. Journal of Food Engineering, 31(2), 263–270.

[fsn31904-bib-0034] Paul, H. (2012). User's manual: Javalytics degree of roast analyzer: Madison Instruments Inc Retrieved from http://www.javalytics.com/wpcontent/uploads/2012/06/JAVALYTICS-JAV-RDA-H-HANDHELD.pdf

[fsn31904-bib-0035] Poltronieri, P. , & Rossi, F. (2016). Review: Challenges in specialty coffee processing and quality assurance. Challenges, 7(19), 22.

[fsn31904-bib-0036] Rodrigues, N. P. , & Bragagnolo, N. (2013). Identification and quantification of bioactive compounds in coffee brews by HPLC‐DAD‐MSn. Journal of Food Composition and Analysis, 32, 105–115.

[fsn31904-bib-0037] Rosa, J. S. D. , Freitas‐Silva, O. , Rouws, J. R. C. , Moreira, I. G. D. S. , Novaes, F. J. M. , Azevedo, D. D. A. , Schwab, N. , Godoy, R. L. D. O. , Eberlin, M. N. , & Rezende, C. M. D. (2016). Mass spectrometry screening of Arabica coffee roasting: A non‐target and non‐volatile approach by EASI‐MS and ESI‐MS. Food Research International, 89, 967–975. 10.1016/j.foodres.2016.03.021

[fsn31904-bib-0127] Sherge, K. T. (2017). Biophysical control of coffee quality: the case of southwestern Ethiopia. Thesis submitted in fulfilment of the requirement of the Degree of Doctor (PhD) in Applied Biological Sciences, Department of Applied Analytical and Physical Chemistry, Isotope Bioscience Laboratory -ISOFYS, Faculty of Bioscience Engineering, Ghent University, Gent, Belgium, Academic year 2016-2017 PhD thesis, 177.

[fsn31904-bib-0038] Soares, C. D. , Cunha, S. , & Fernandes, J. (2006). Determination of acrylamide in coffee and coffee products by GC‐MS using an improved SPE clean‐up. Food Additives and Contaminants, 23(12), 1276–1282. 17118870 10.1080/02652030600889608

[fsn31904-bib-0039] Specialty Coffee Association of America (2015). SCAA protocols ‐ cupping specialty coffee (pp. 7) Retrieved from https://www.scaa.org/PDF/resources/cupping-protocols.pdf

[fsn31904-bib-0040] Sualeh, A. , & Mekonnen, N. (2015). In Kirub A. (Ed.), Manual for coffee quality laboratory. Ethiopian Institute of Agricultural Research Retrieved from http://publication.eiar.gov.et:8080/xmlui/handle/123456789/152?show=full

[fsn31904-bib-0041] Tai, J. , Cheung, S. , Chan, E. , & Hasman, D. (2010). Antiproliferation effect of commercially brewed coffees on human ovarian cancer cells in vitro. Nutrition and Cancer, 62(8), 1044–1057. 21058192 10.1080/01635581.2010.492083

[fsn31904-bib-0042] Vignoli, J. , Viegas, M. , Bassoli, D. , & Benassi, M. (2014). Roasting process affects differently the bioactive compounds and the antioxidant activity of arabica and robusta coffees. Food Research International, 61, 279–285.

[fsn31904-bib-0043] Wang, X. , & Lim, L. T. (2014). Effect of roasting conditions on carbon dioxide degassing behavior in coffee. Food Research International, 61, 144–151.

