# Supplementary material for: Effects of coffee roasting technologies on cup quality and bioactive compounds of specialty coffee beans
Source: Food Sci Nutr. 2020 Oct 8;8(11):6120–30. doi: 10.1002/fsn3.1904 (PMC7684626; doi:10.1002/fsn3.1904)
Supplement: Supplementary file 1 — Appendix [file FSN3-8-6120-s001.docx]

**Appendix A. HPLC Chromatograms of Sample Raw Coffee Beans**


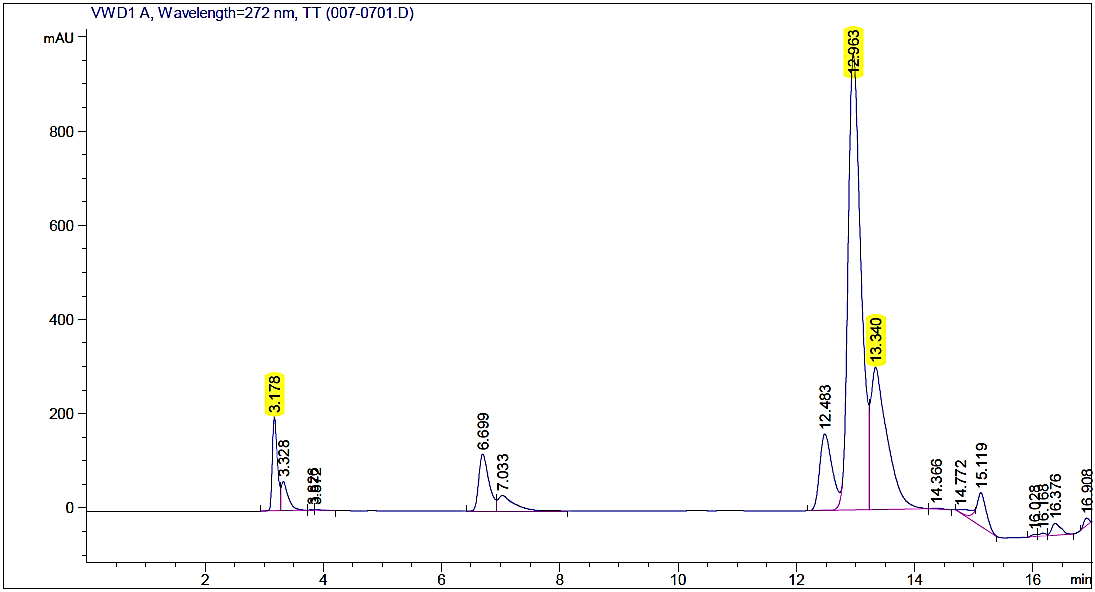


Yirgacheffe


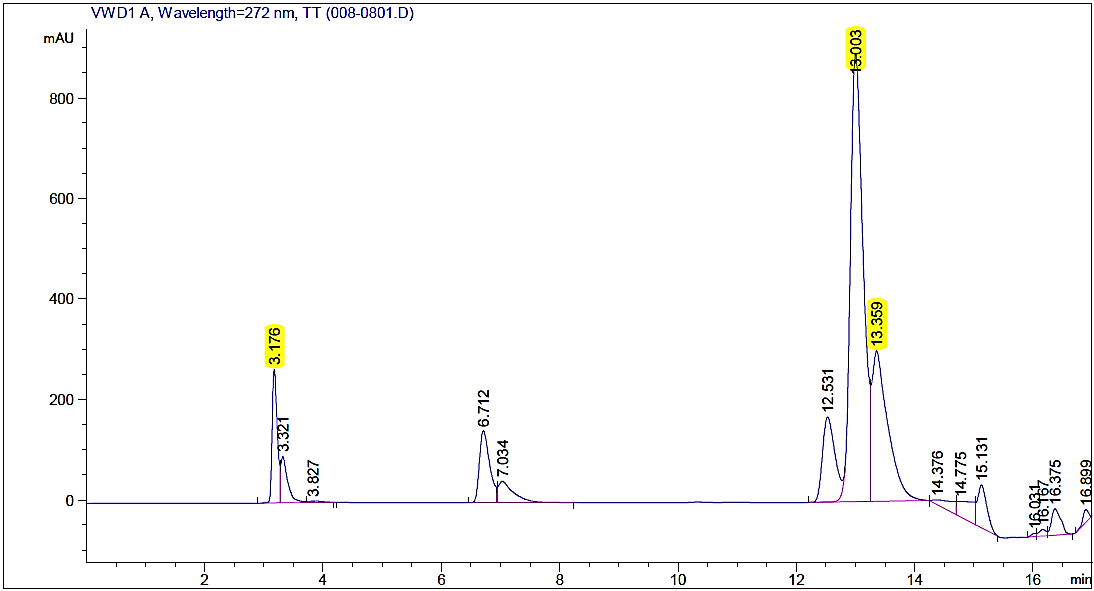

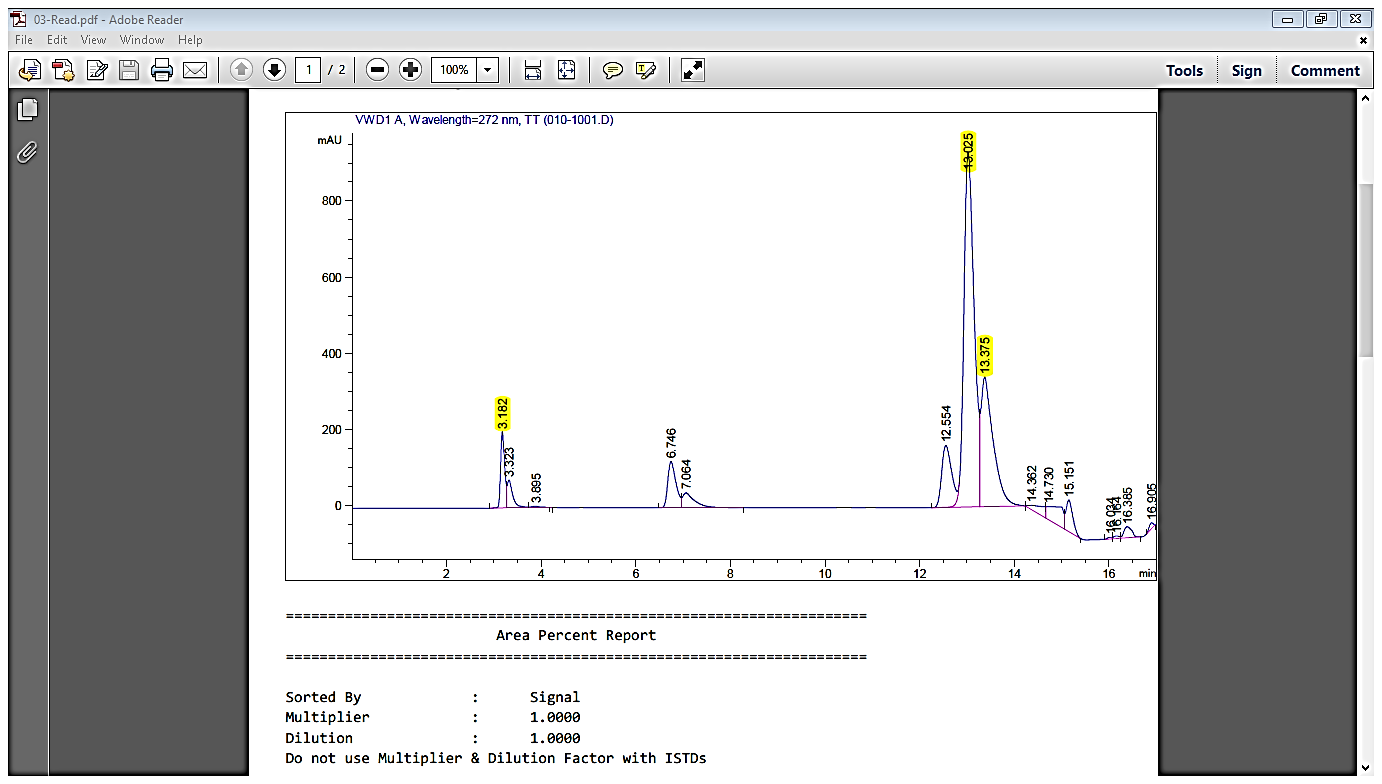


Sidama

Harar

**Appendix B. Calibration Curves for the HPLC Analysis**

Caffeine

Conc. (mg/g)

Peak Area

Trigonelline

Conc. (mg/g)

Peak Area

CGAs

Conc. (mg/g)

Peak Area

**Appendix C. HPLC Chromatograms of Roasted Coffee Beans**


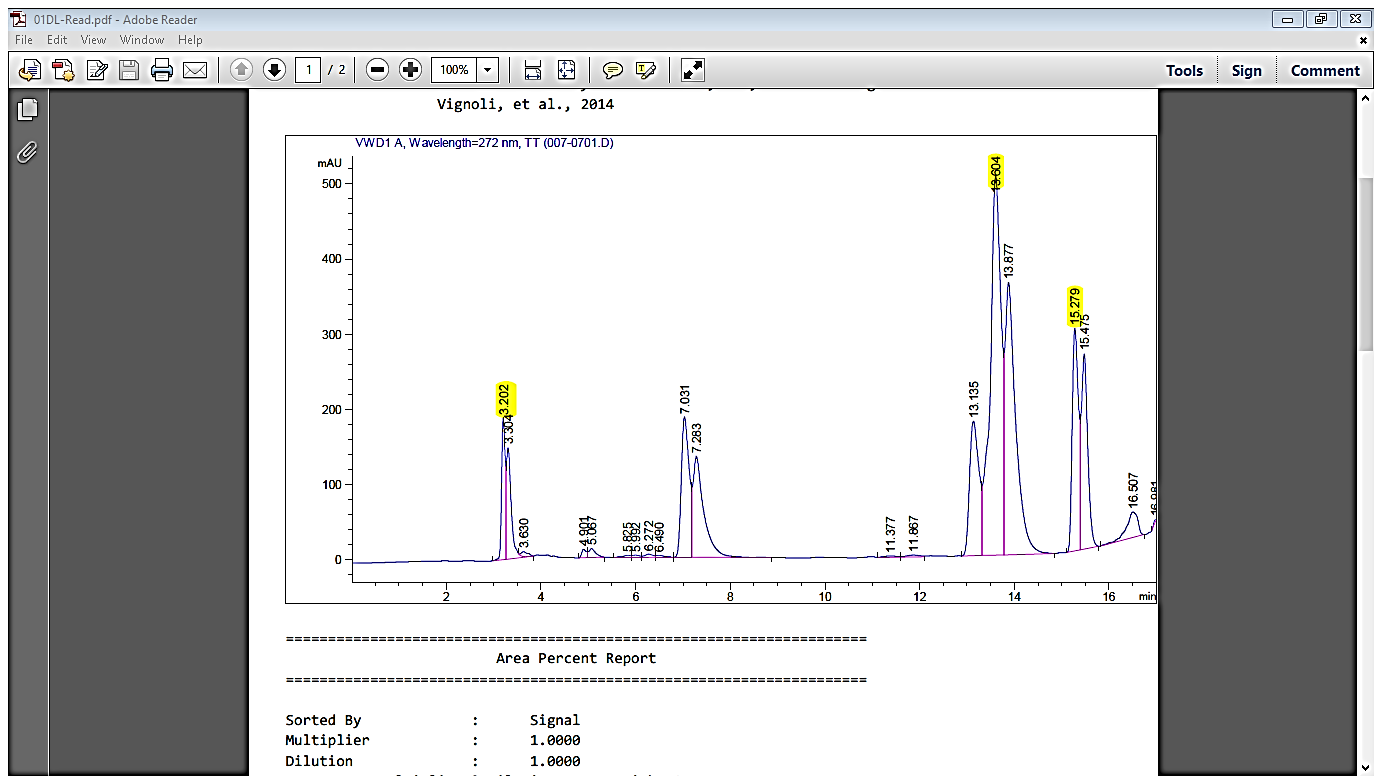

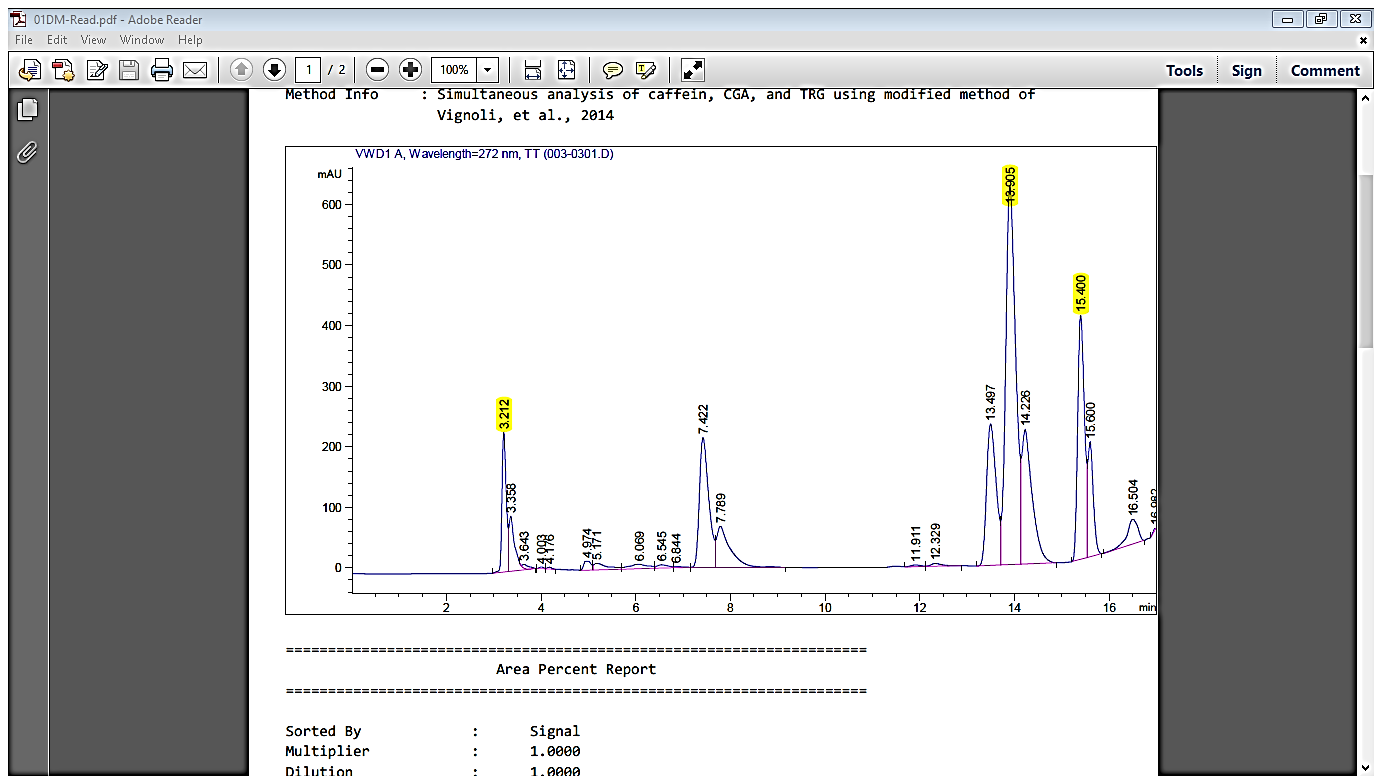


01DM

01DL


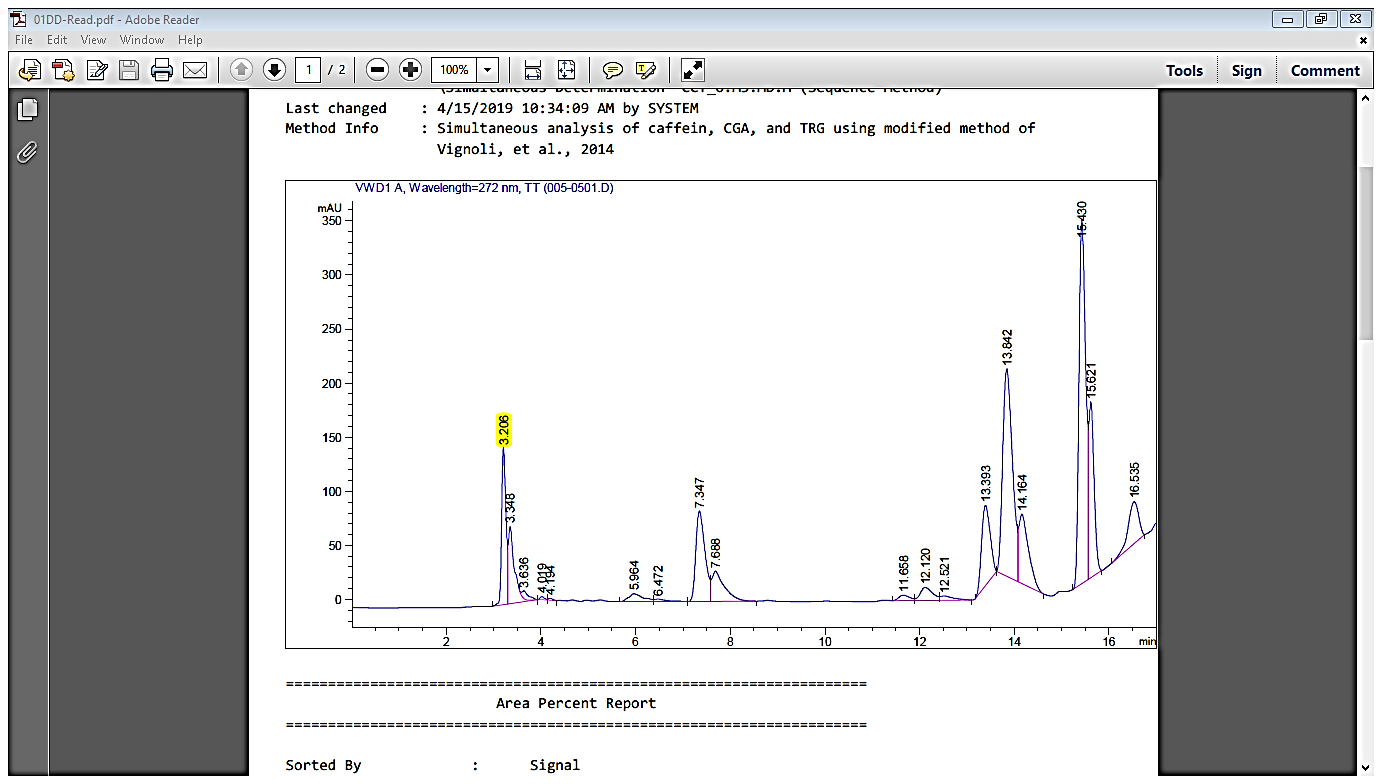

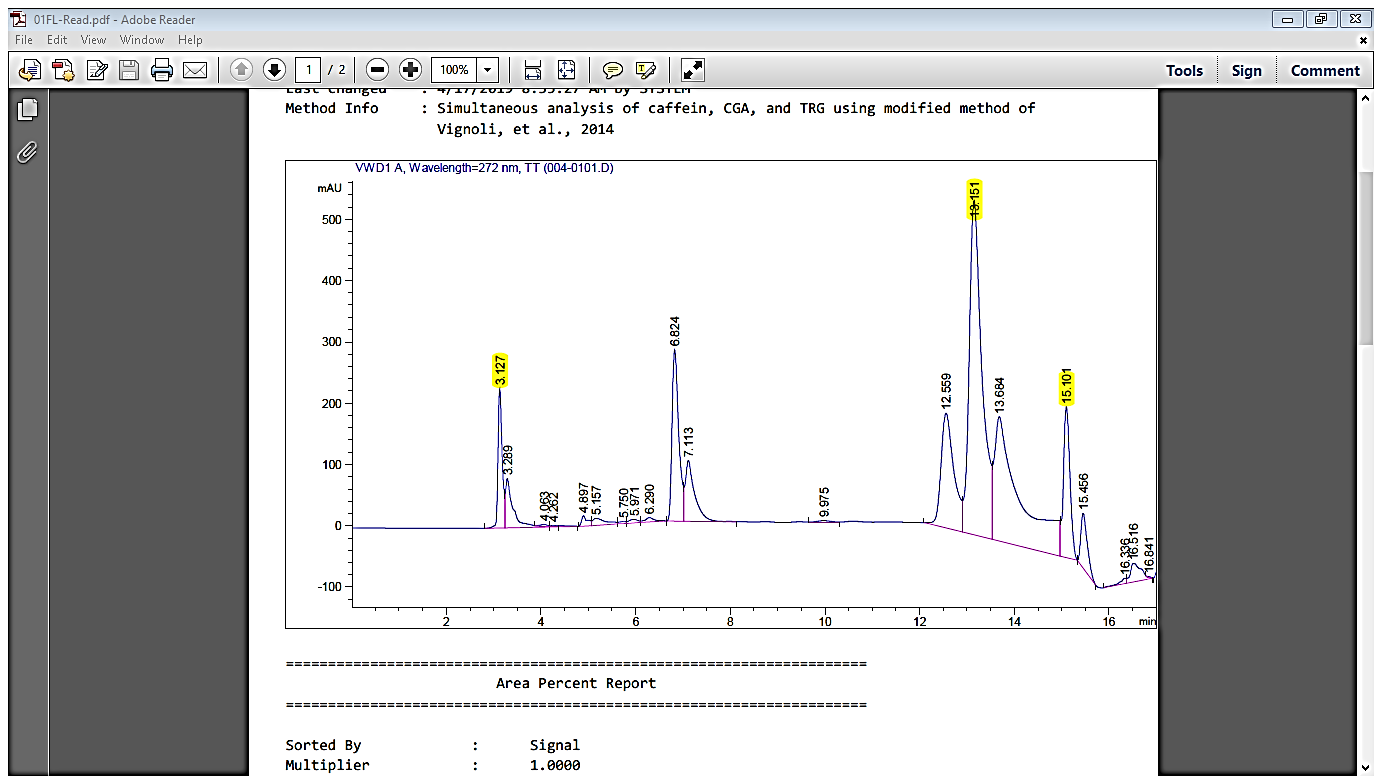


01FL

01DD

1. 01DL, 01DM, and 01DD are sample Yirgacheffe coffees roasted using drum roaster at light, medium, and dark degree of roast, respectively.
2. 01FL is sample Yirgacheffe coffee roasted using fluidized bed roaster at light degree of roast.


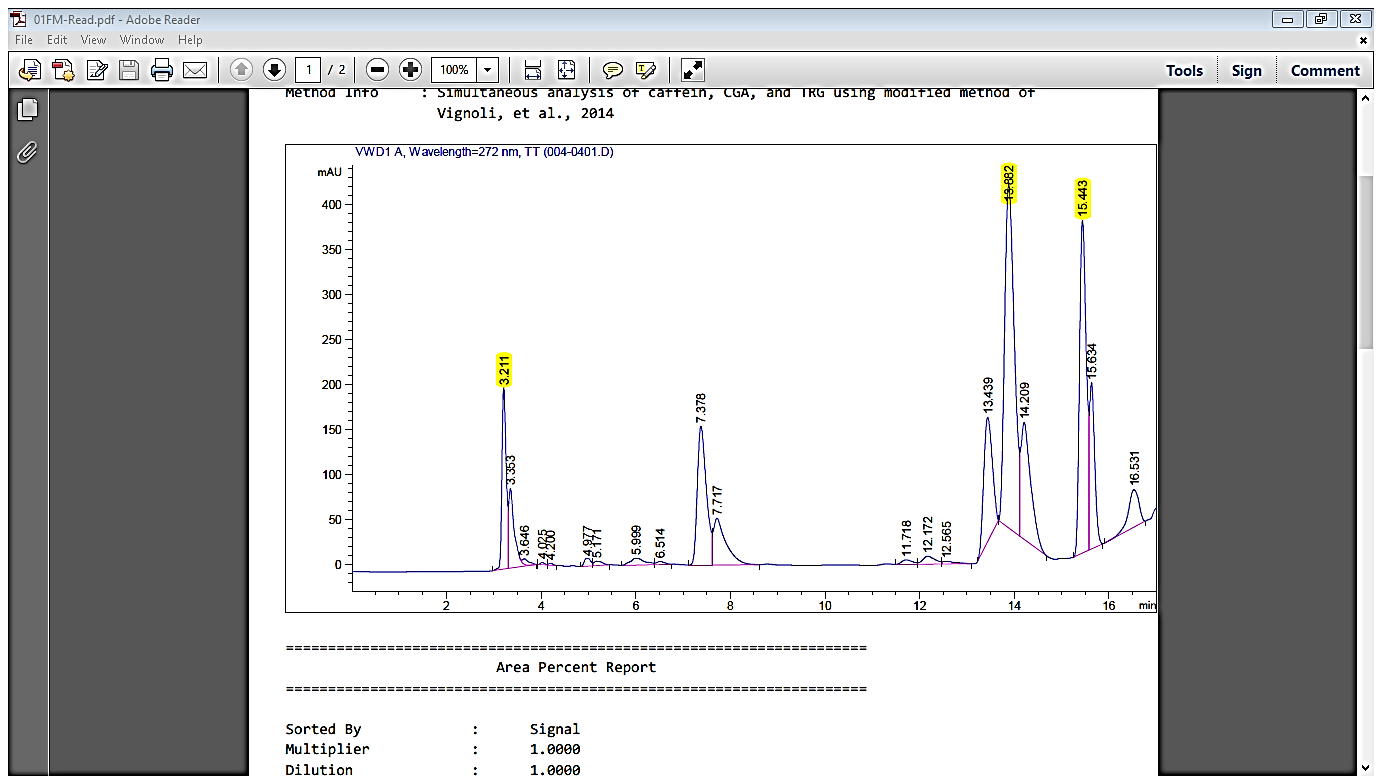

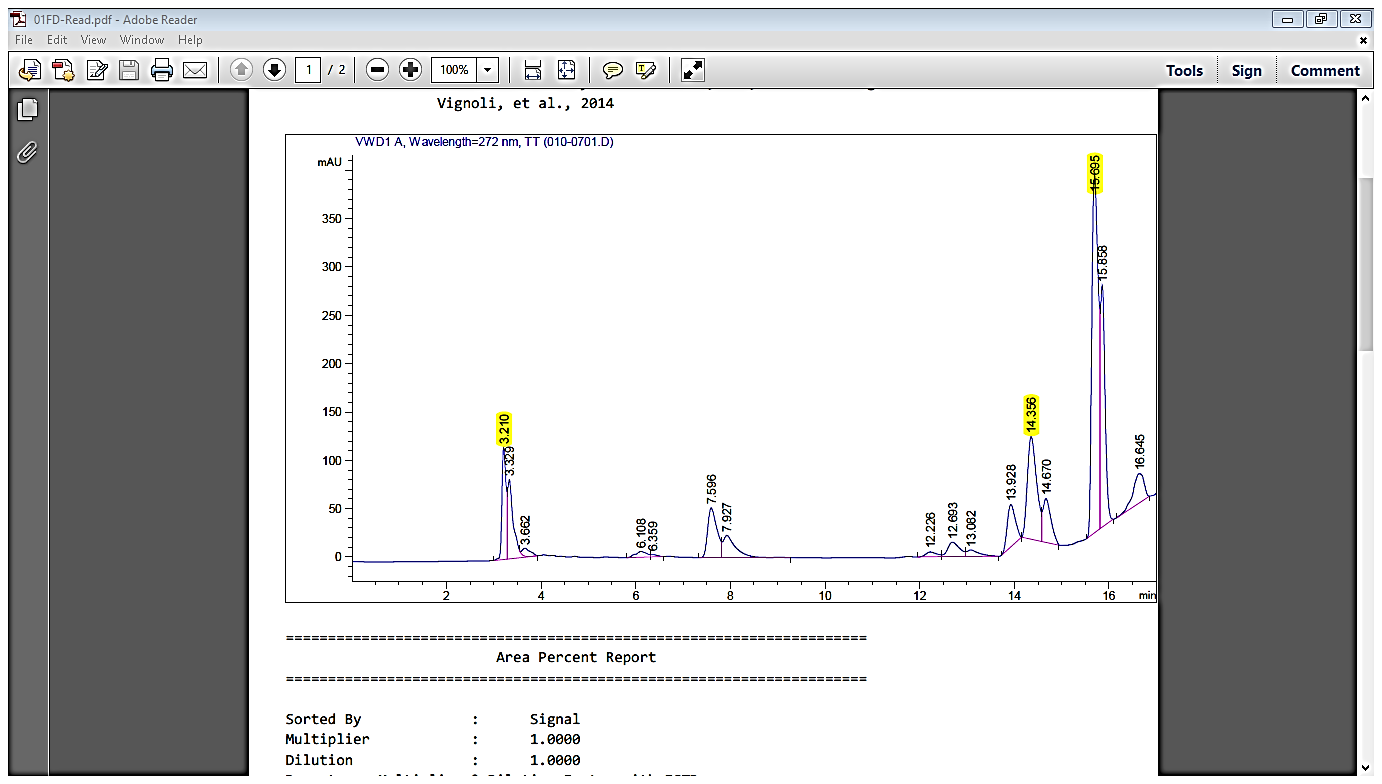


01FD

01FM


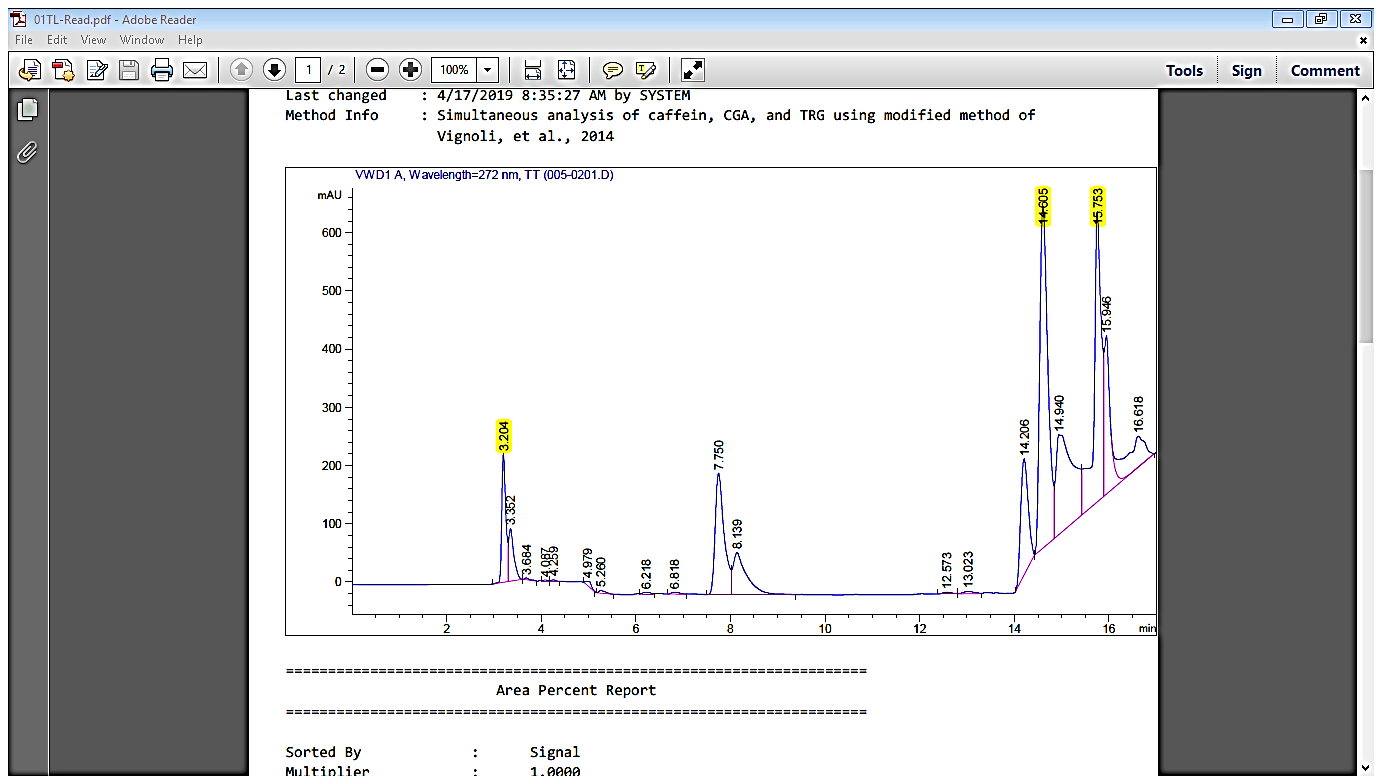

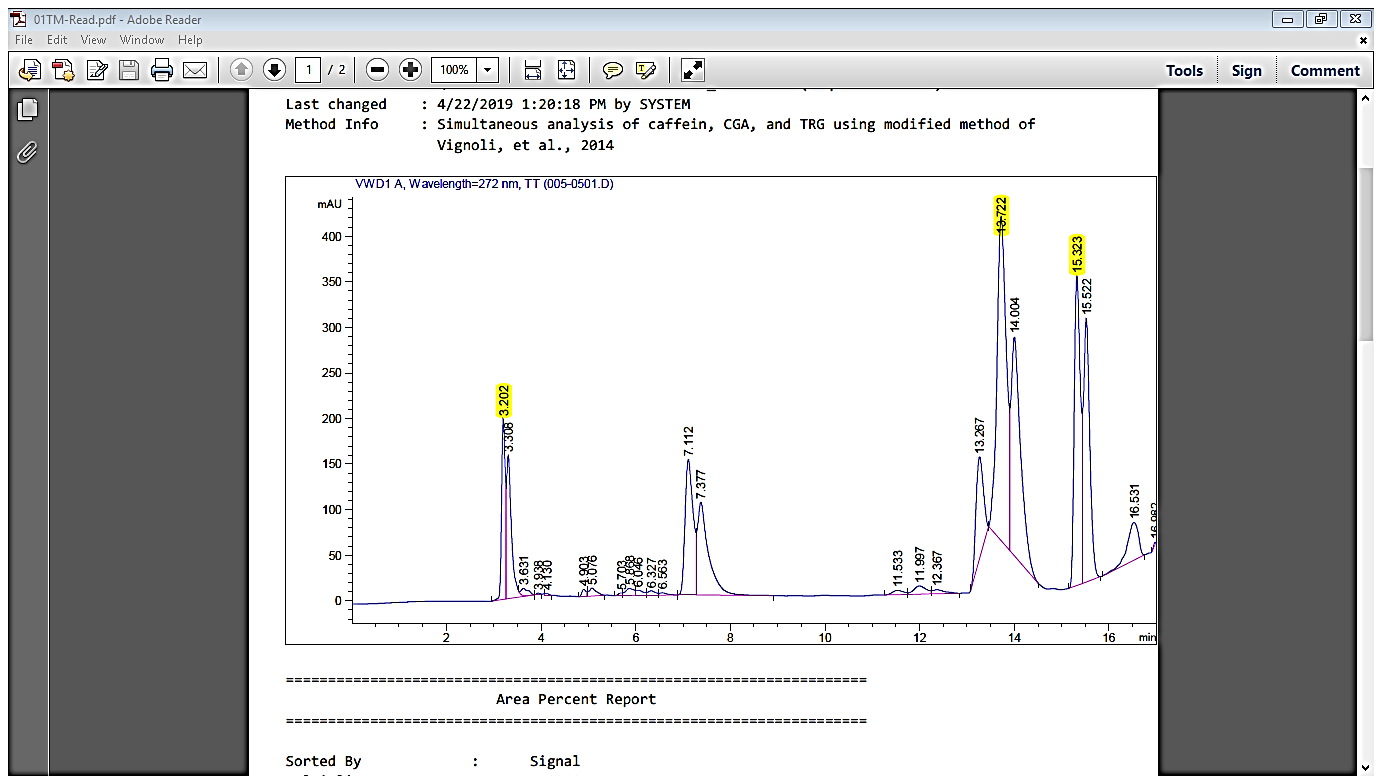


01TM

01TL

1. 01FM and 01FD are sample Yirgacheffe coffees roasted using fluidized bed roaster at medium and dark degree of roast, respectively.
2. 01TL and 01TM are sample Yirgacheffe coffees roasted using traditional roaster at light and medium degree of roast, respectively.


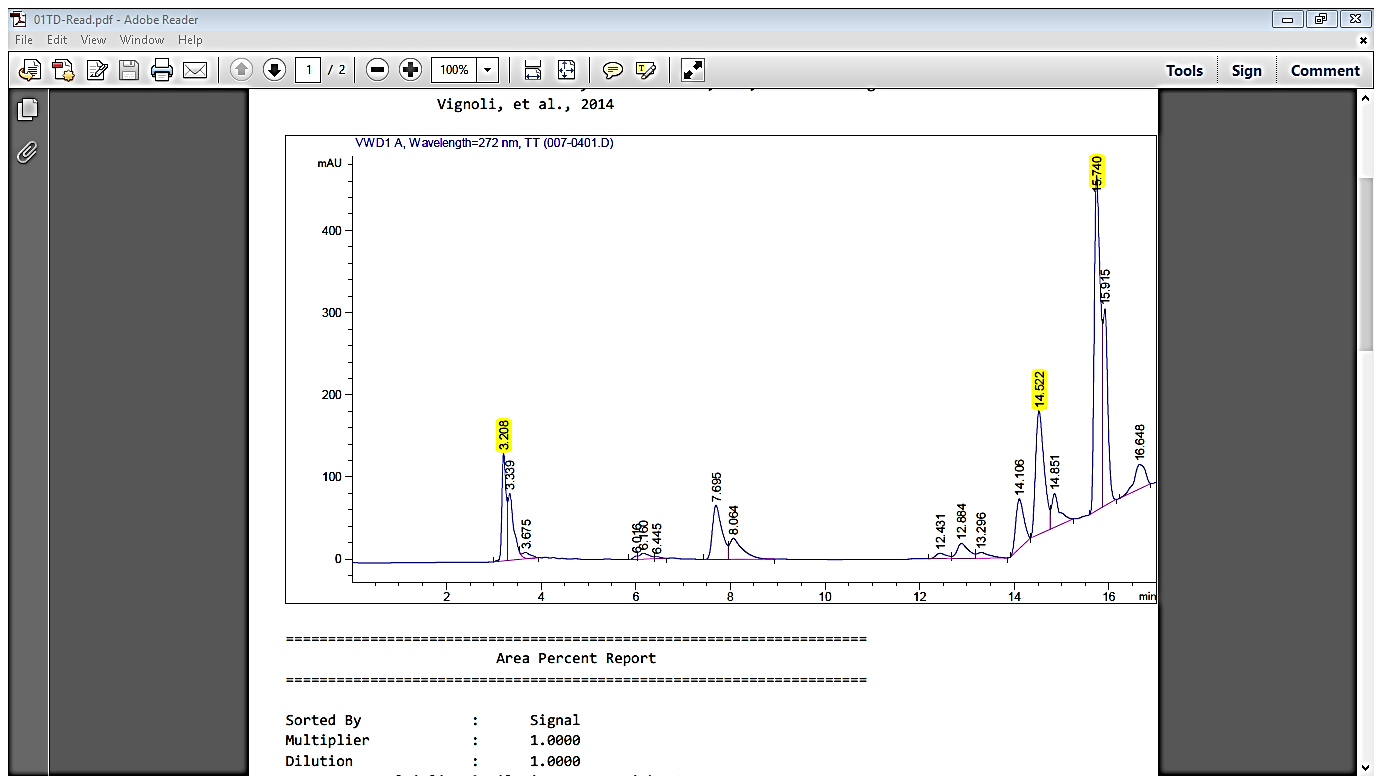

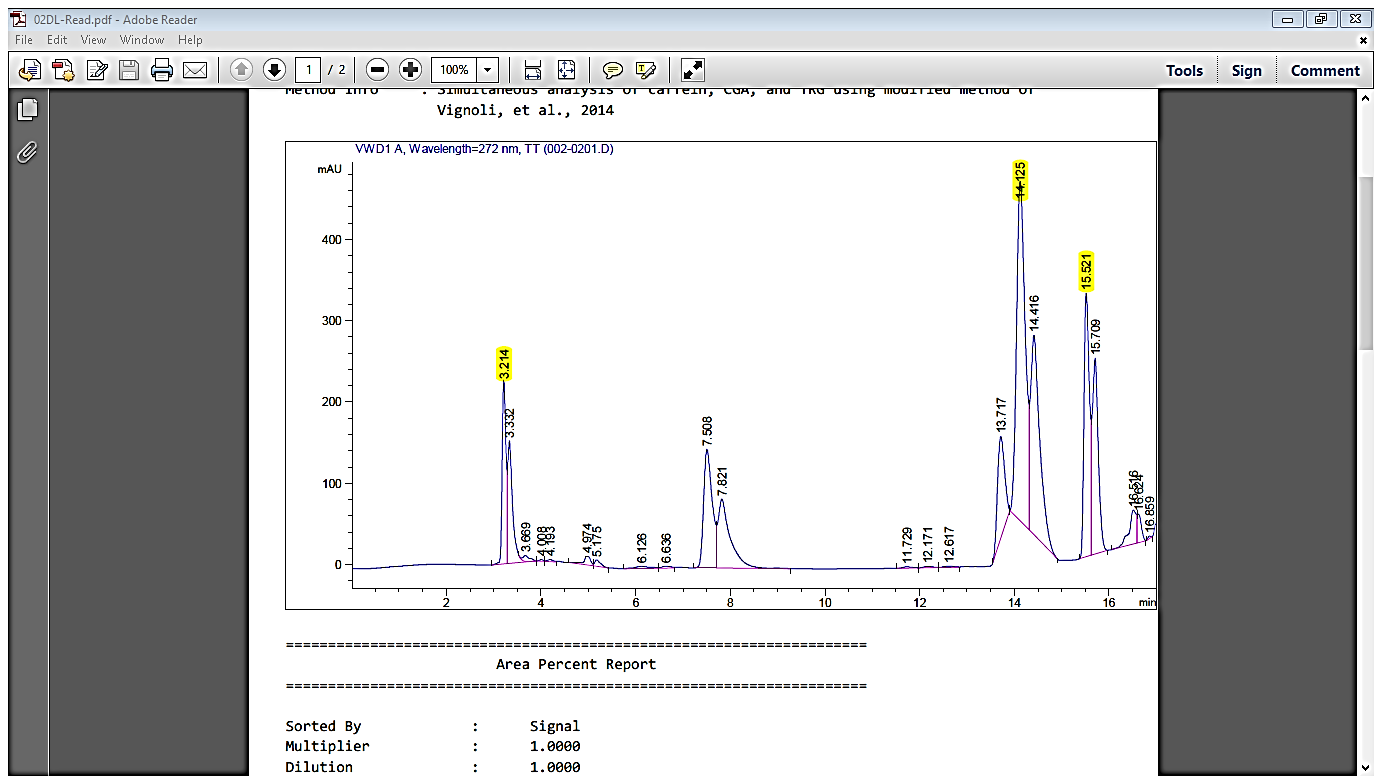


02DL

01TD


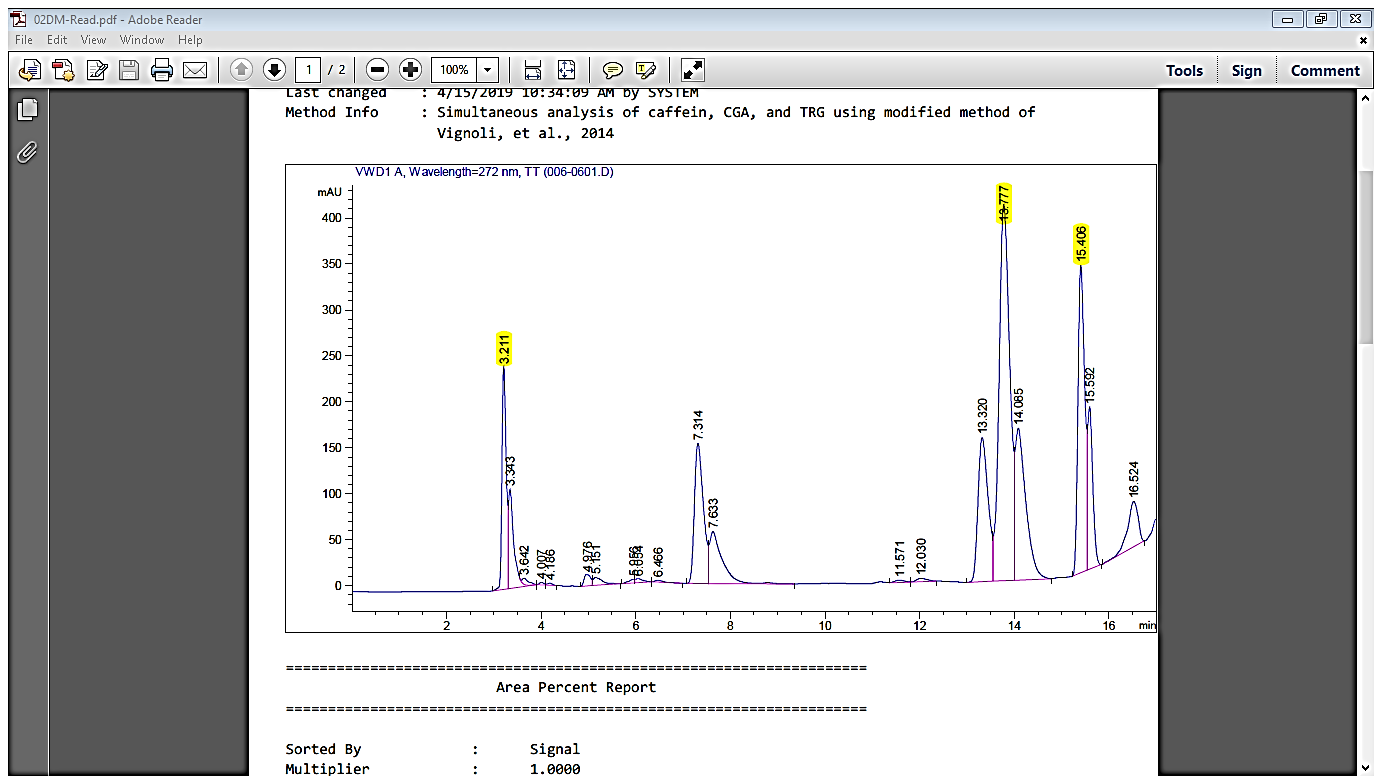

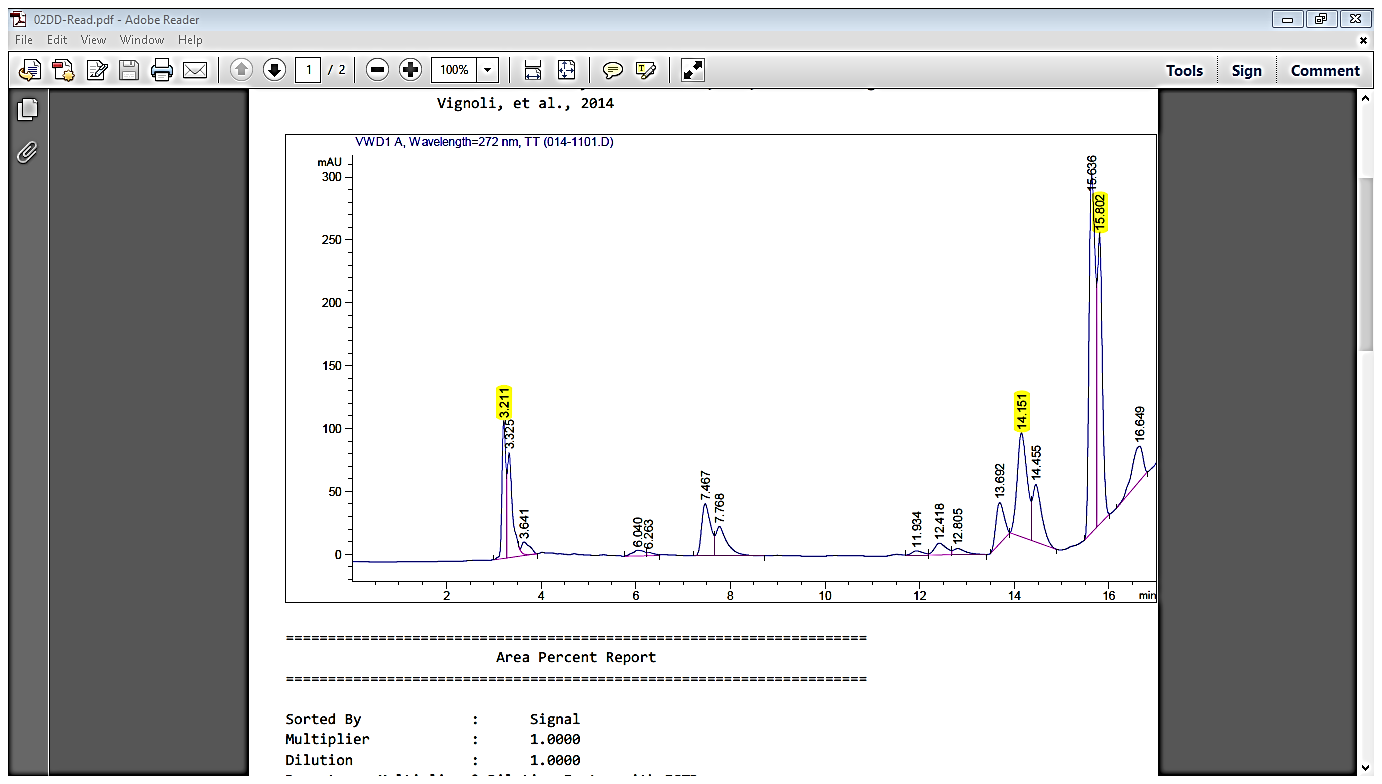


02DD

02DM

1. 01TD is sample Yirgacheffe coffee roasted using traditional roaster at dark degree of roast.
2. 02DL, 02DM and 02DD are sample Harar coffees roasted using drum roaster at light, medium, and dark degree of roast, respectively.


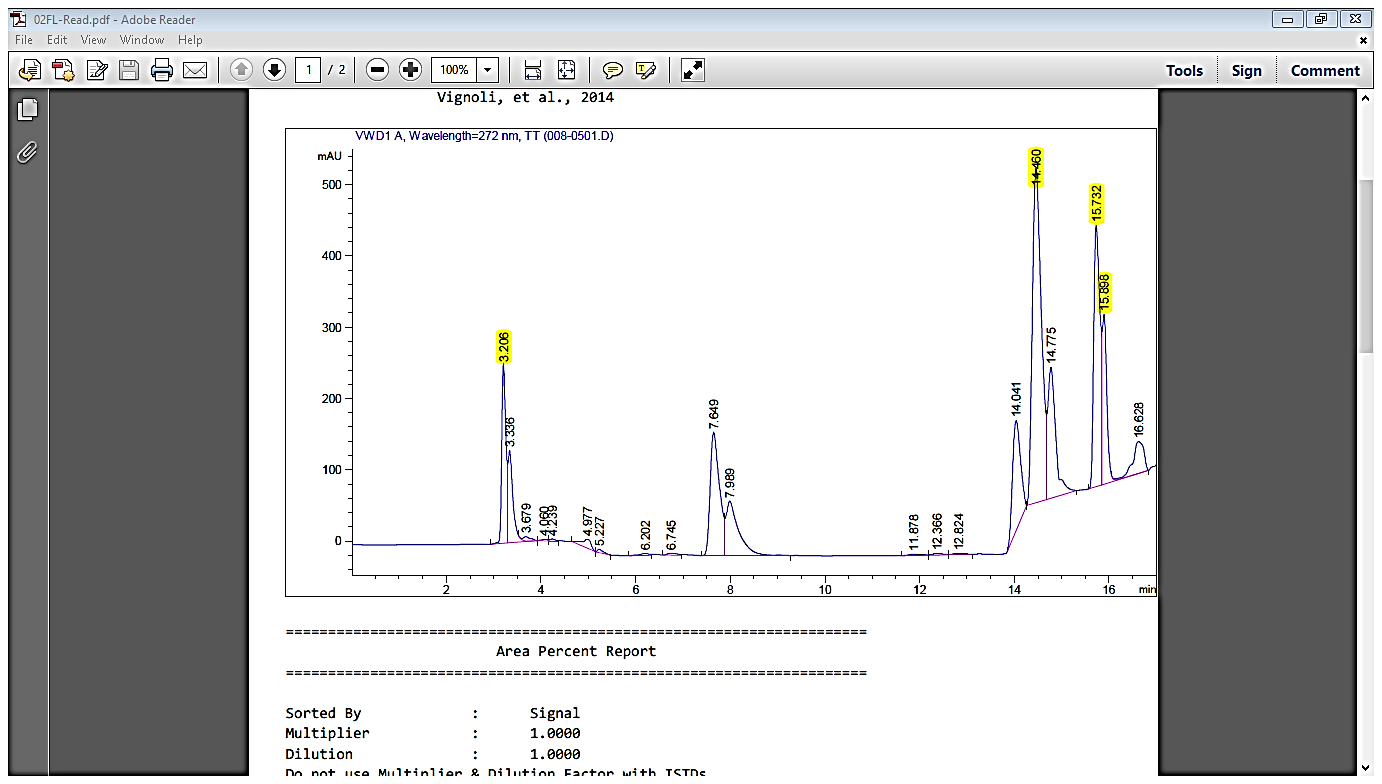

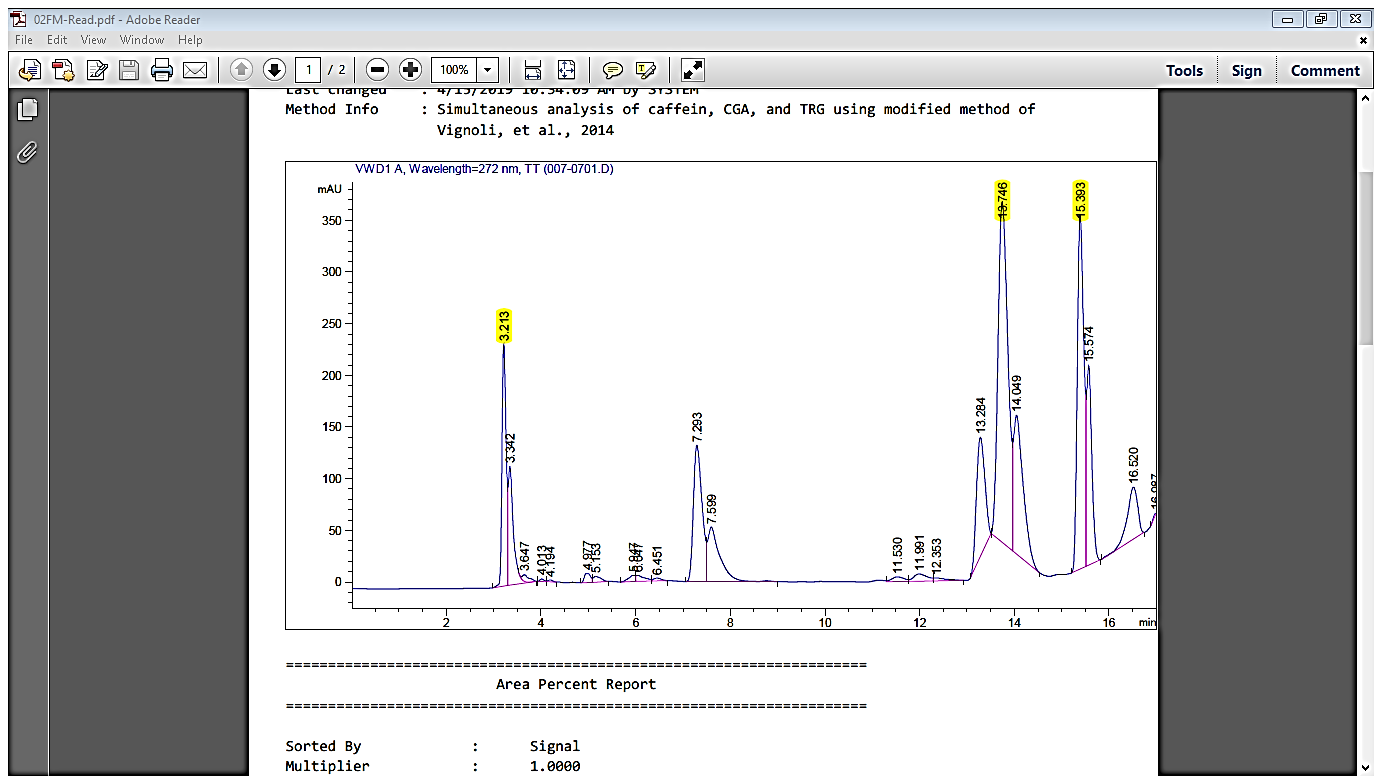


02FL

02FM


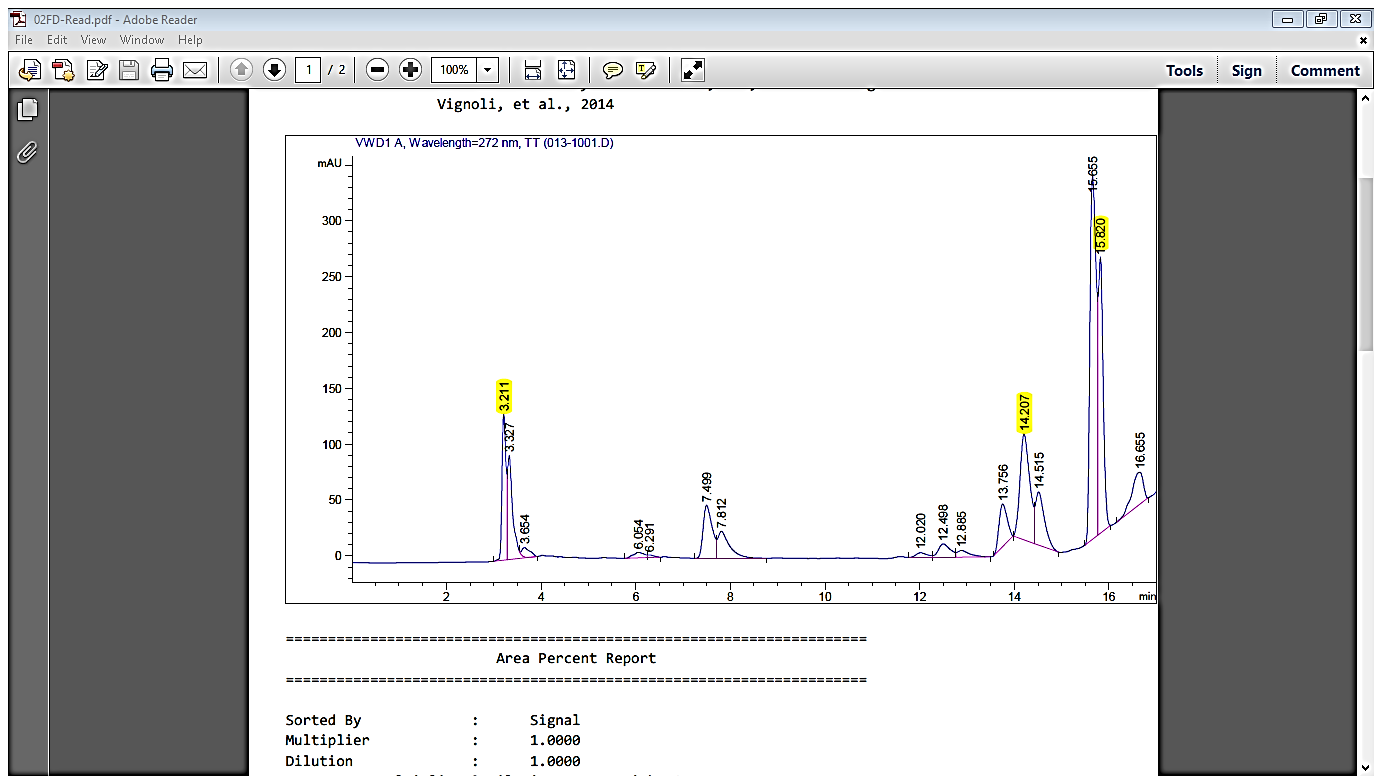

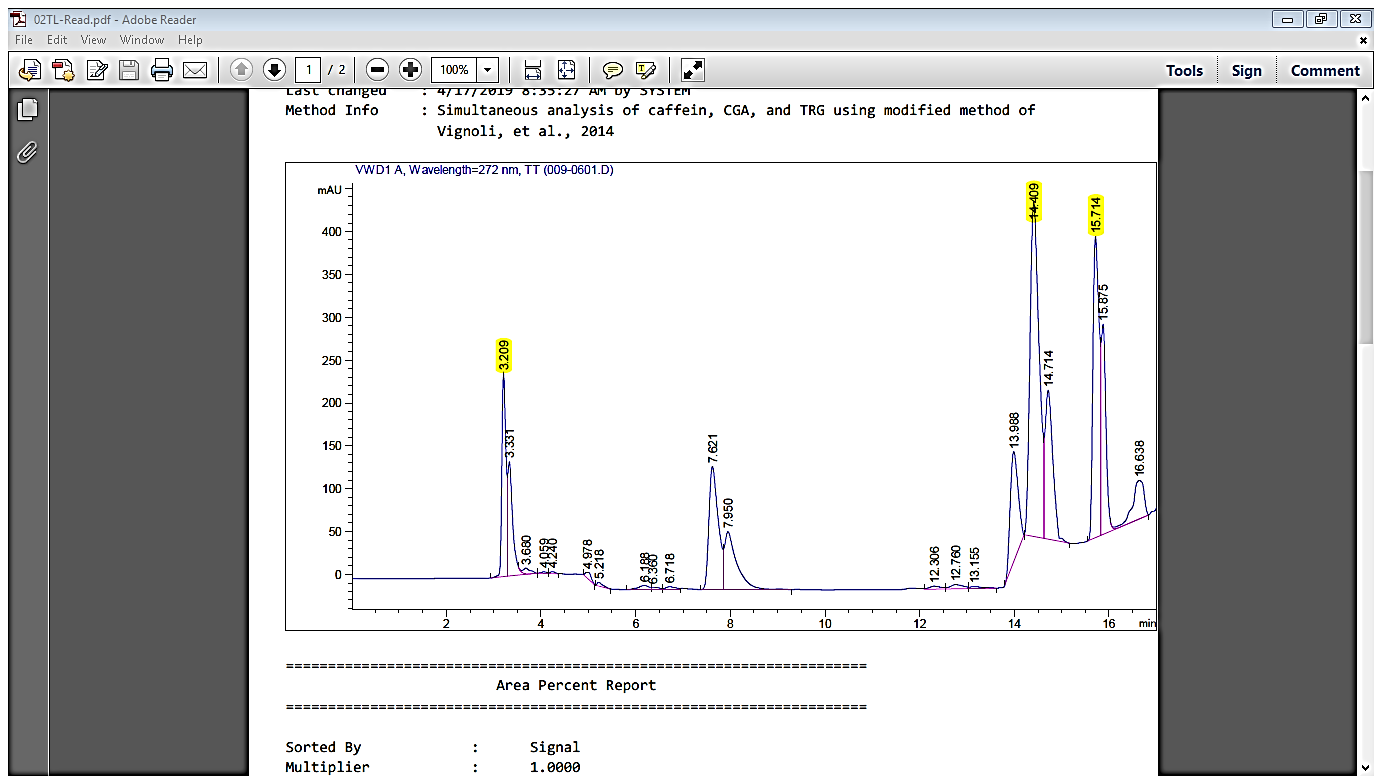


02FD

02TL

1. 02FL, 02FM and 02FD are sample Harar coffees roasted using fluidized bed roaster at light, medium, and dark degree of roast, respectively.
2. 02TL is sample Harar coffee roasted using traditional roaster at light degree of roast.


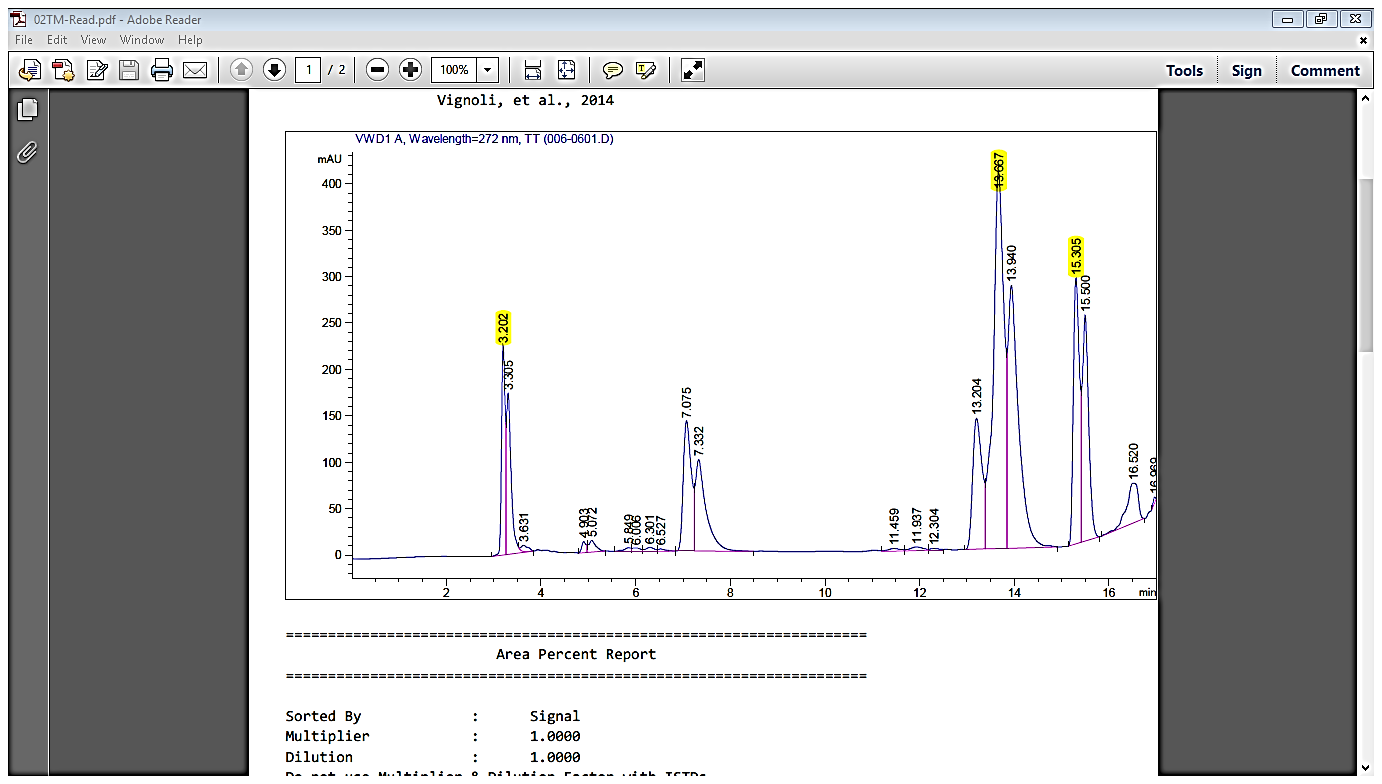

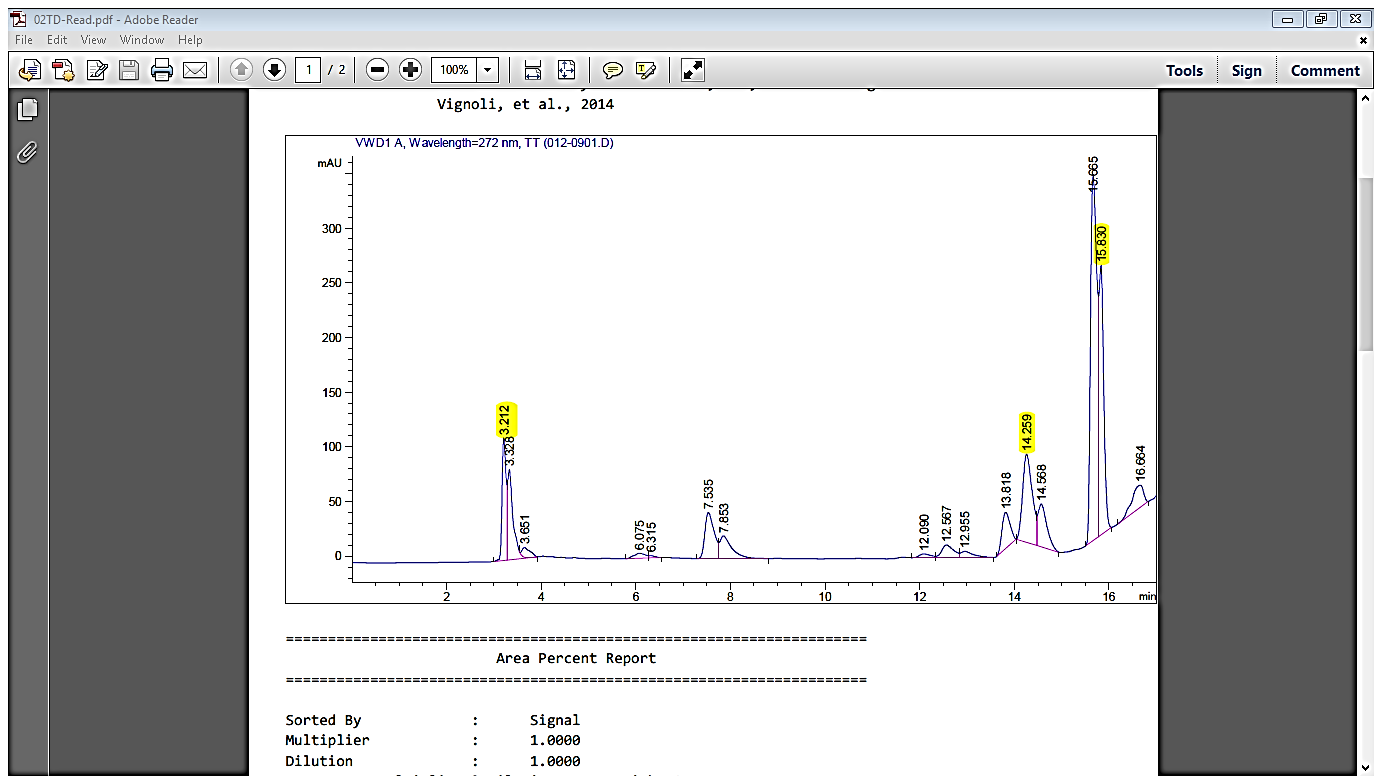


02TD

02TM


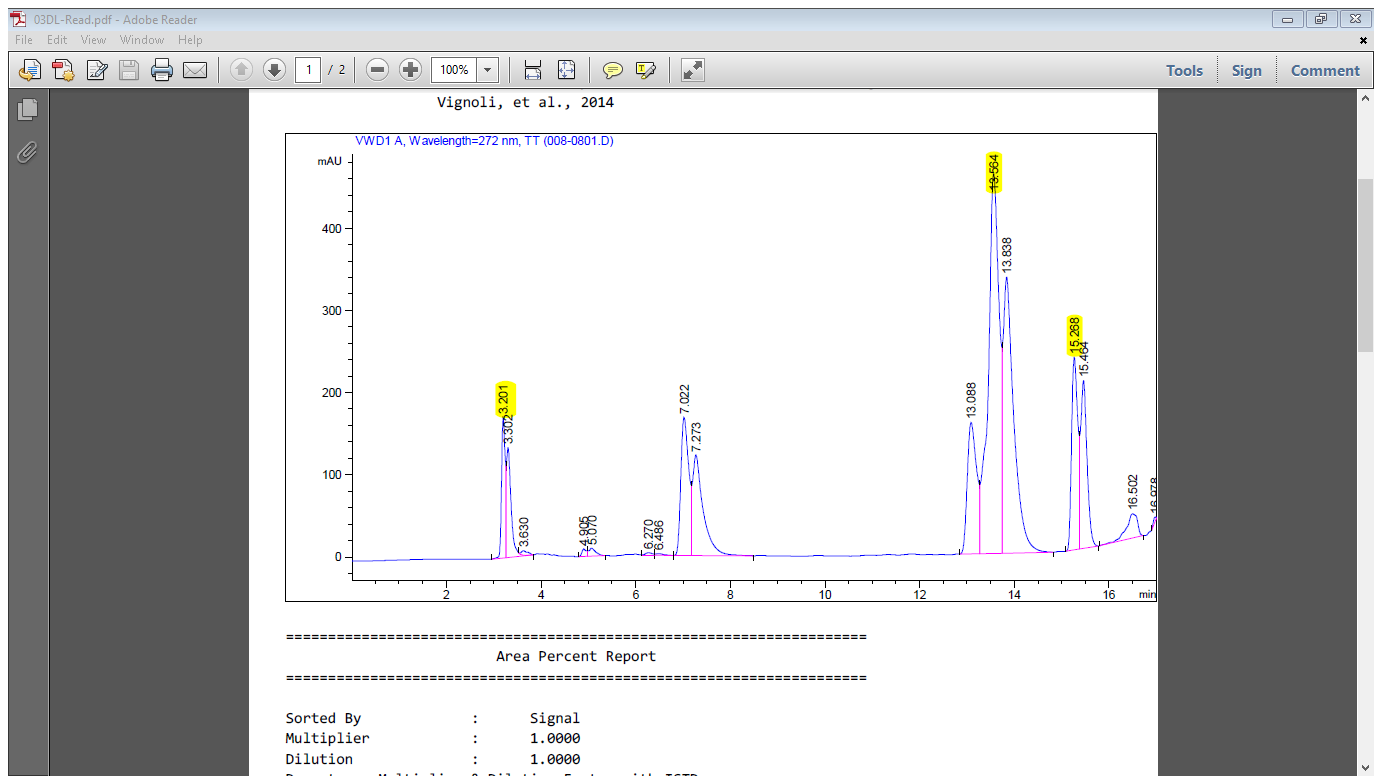

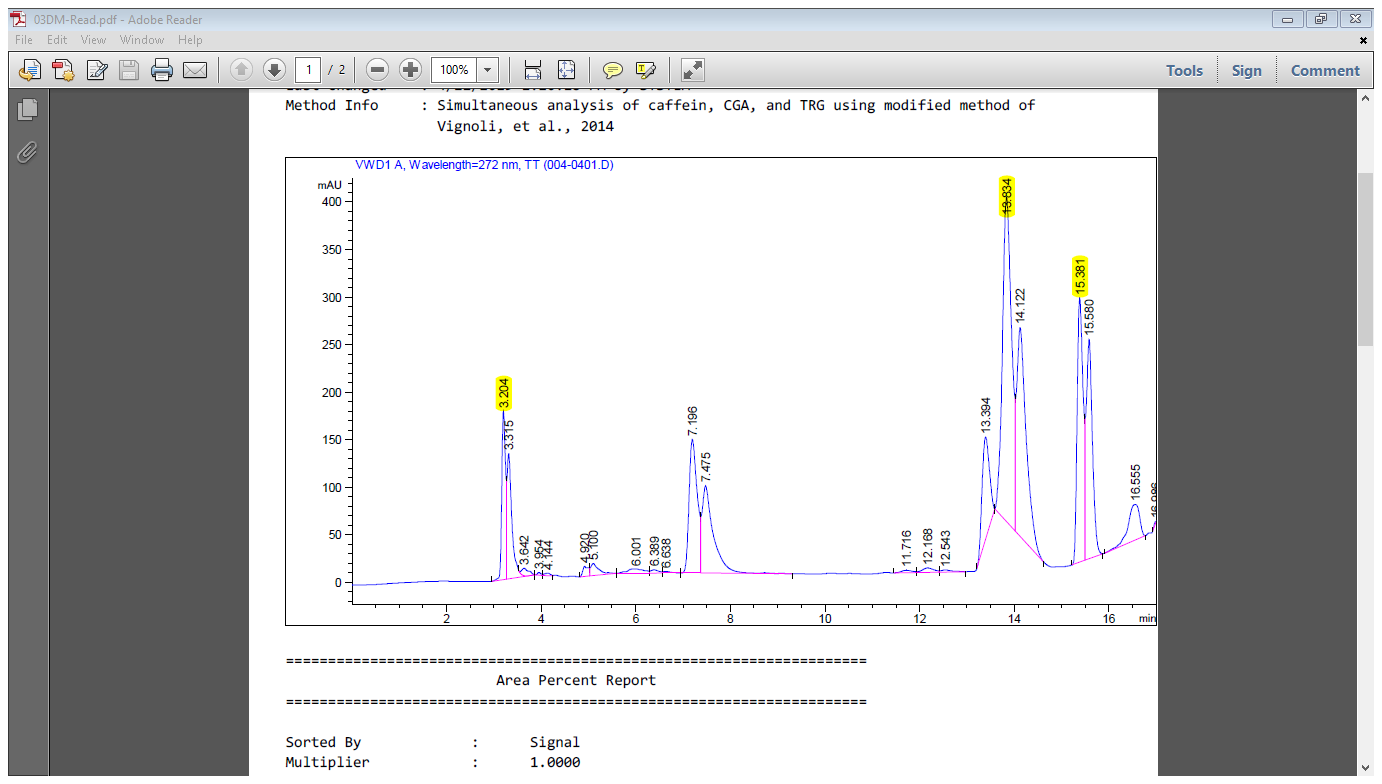


03DM

03DL

1. 02TM and 02TD are sample Harar coffees roasted using traditional roaster at medium and dark degree of roast, respectively.
2. 03DL and 03DM are sample Sidama coffees roasted using drum roaster at light and medium degree of roast, respectively.


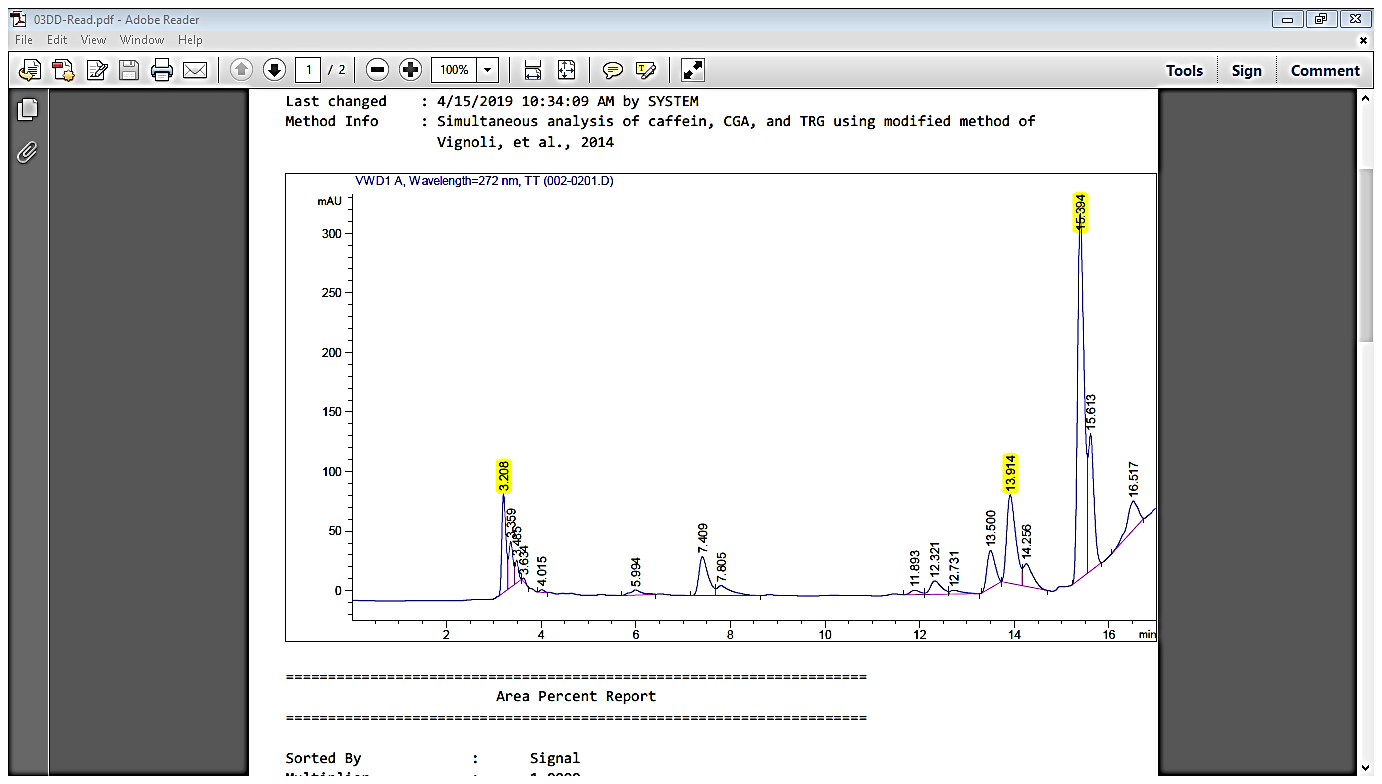

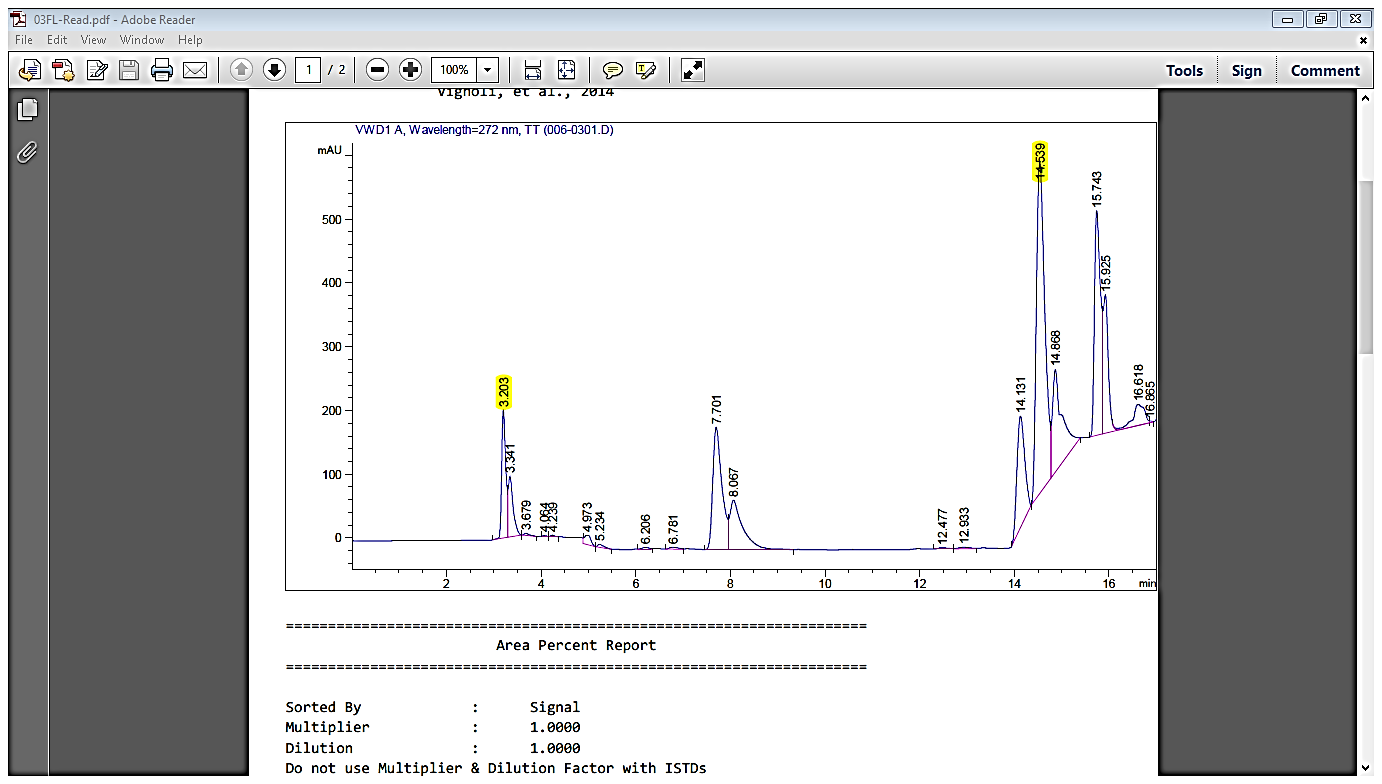


03FL

03DD


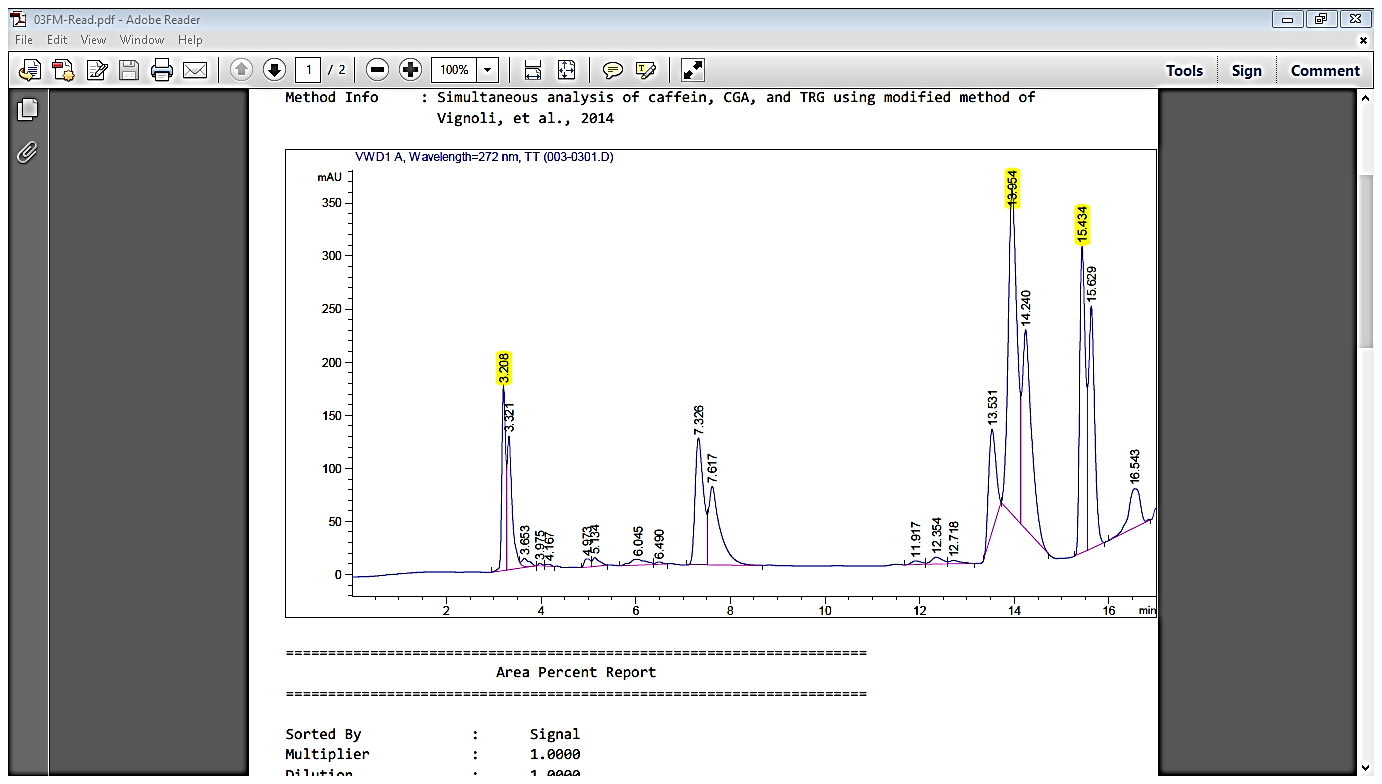

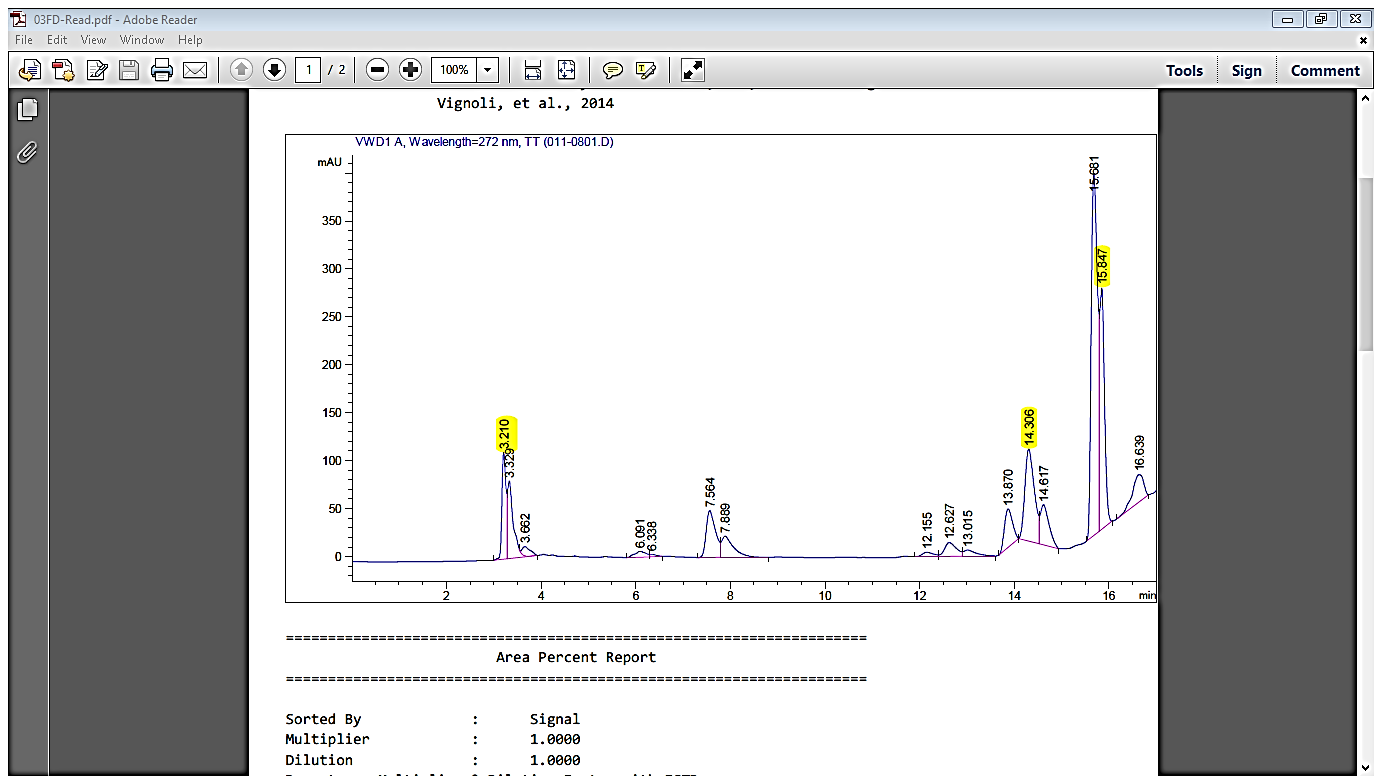


03FD

03FM

1. 03DD is sample Sidama coffee roasted using drum roaster at dark degree of roast.
2. 03FL, 03FM and 03FD are sample Sidama coffees roasted using fluidized bed roaster at light, medium, and dark degree of roast, respectively.

**Appendix D. Photos of Sample Coffee Beans**

1. Raw Sample Coffee Beans


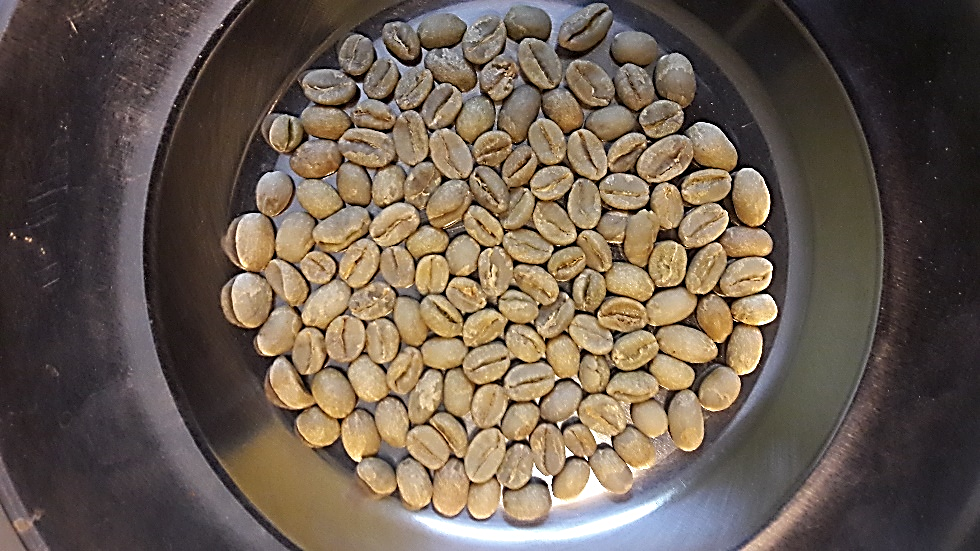

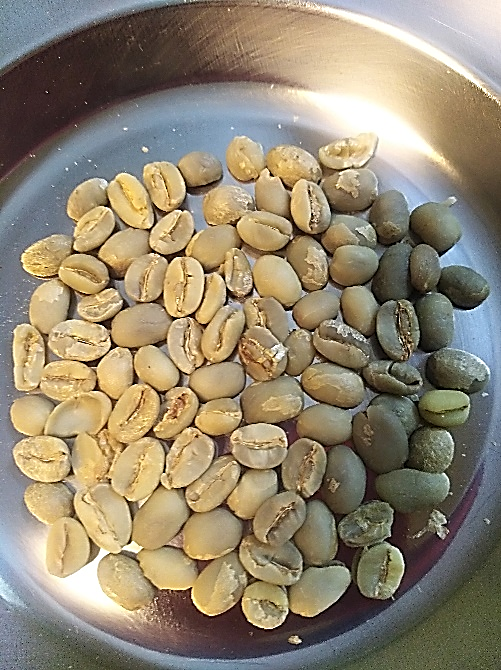

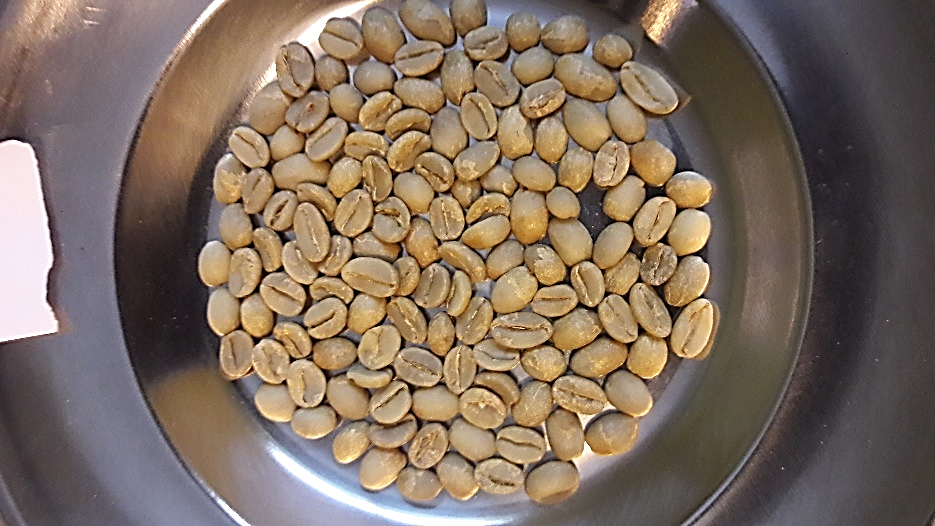


Sidama

Harar

Yirgacheffe

1. Roasted Sample Coffee Beans


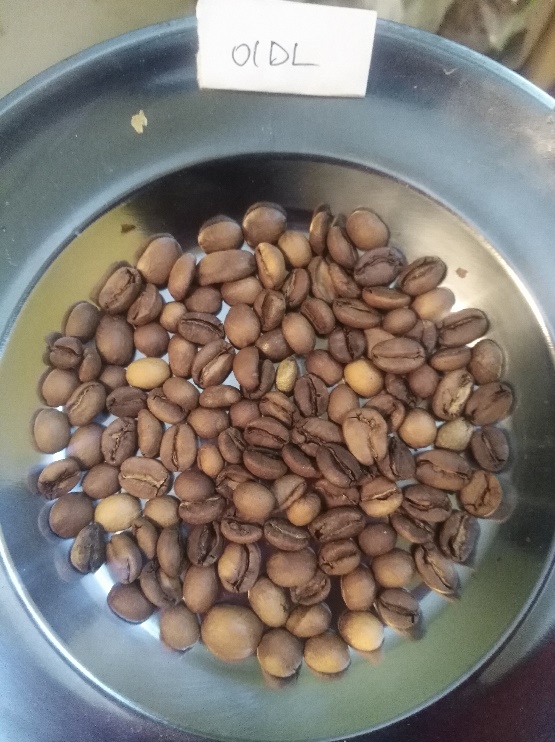

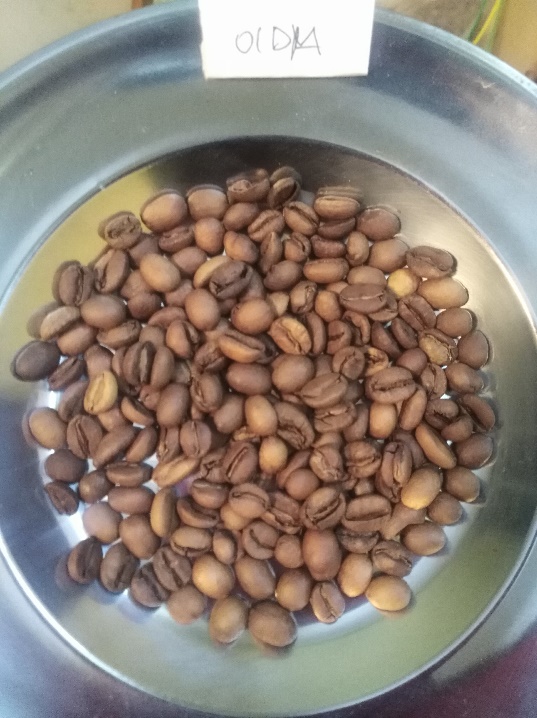

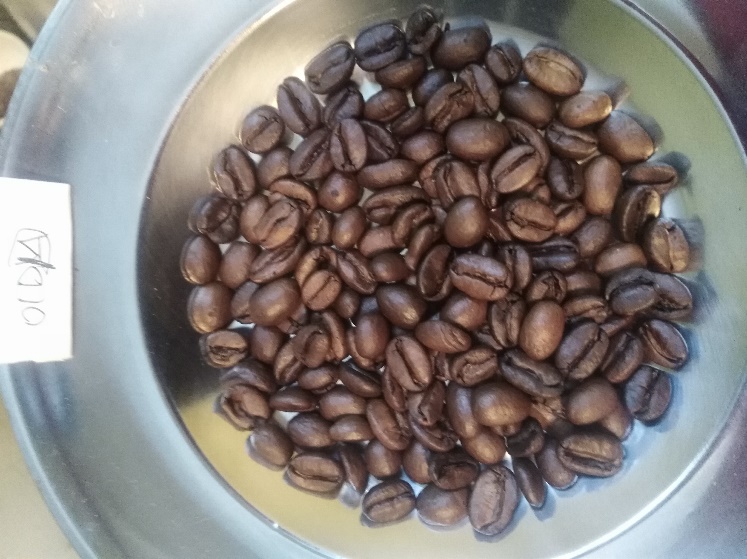


01DD

01DM

01DL


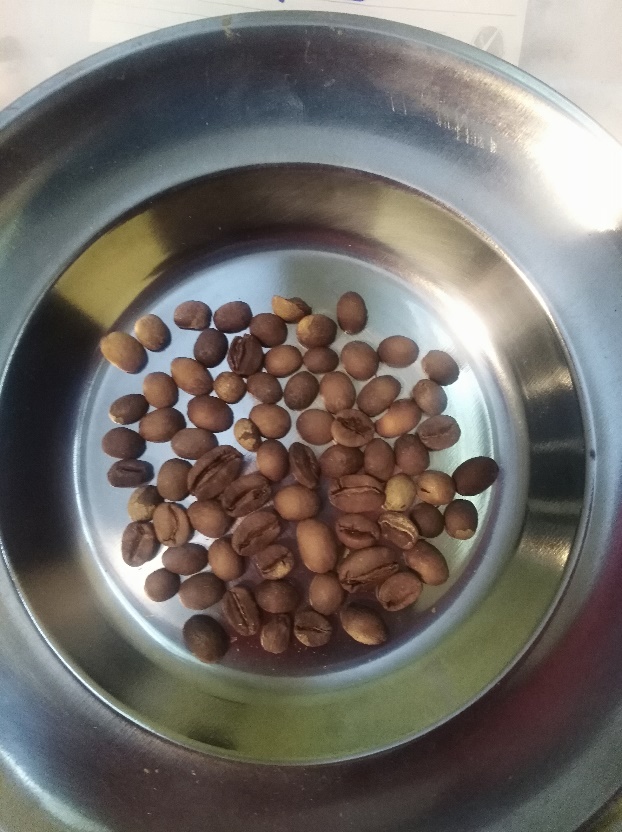

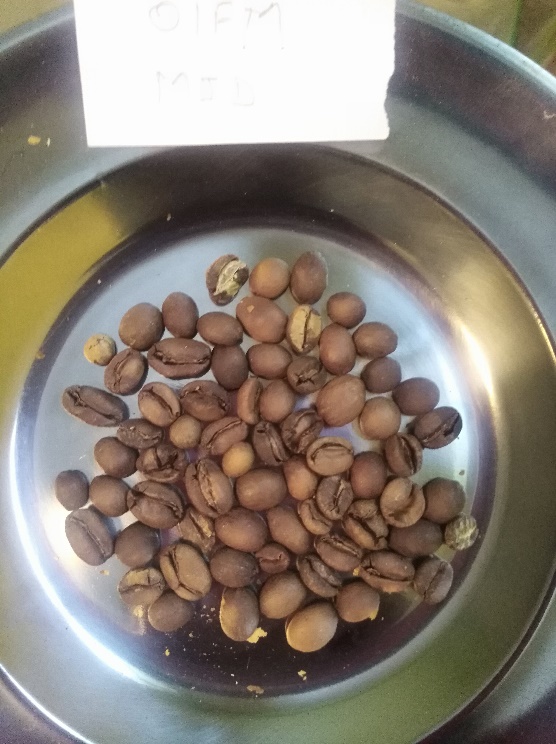

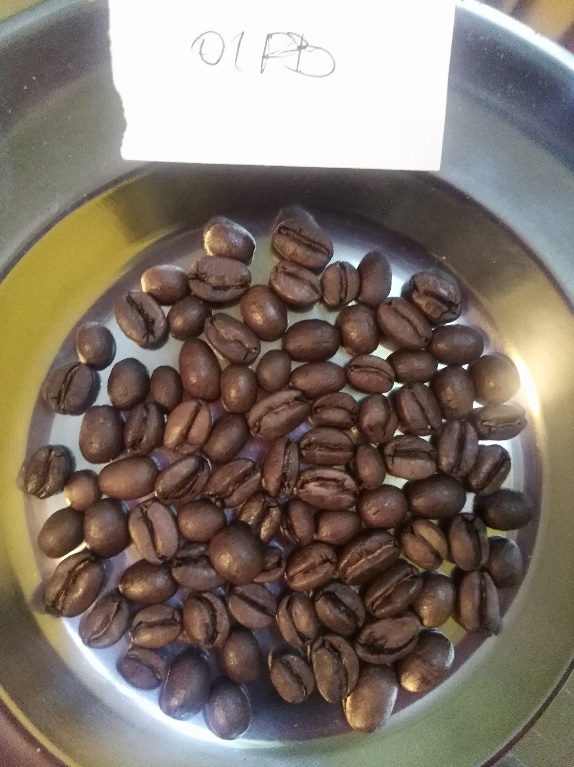


01FD

01FM

01FL


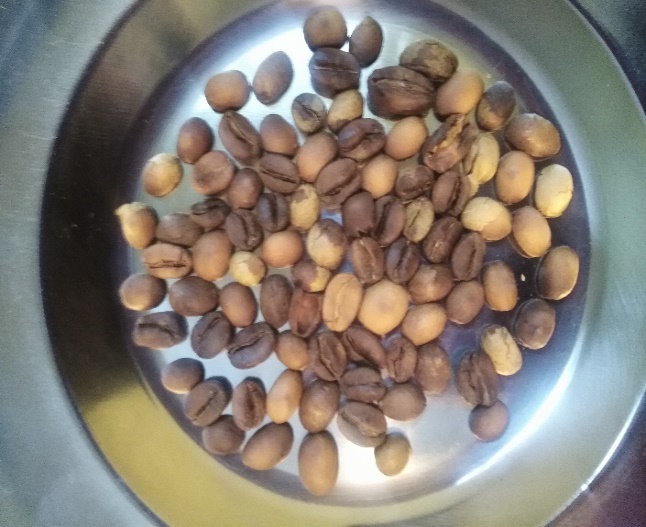

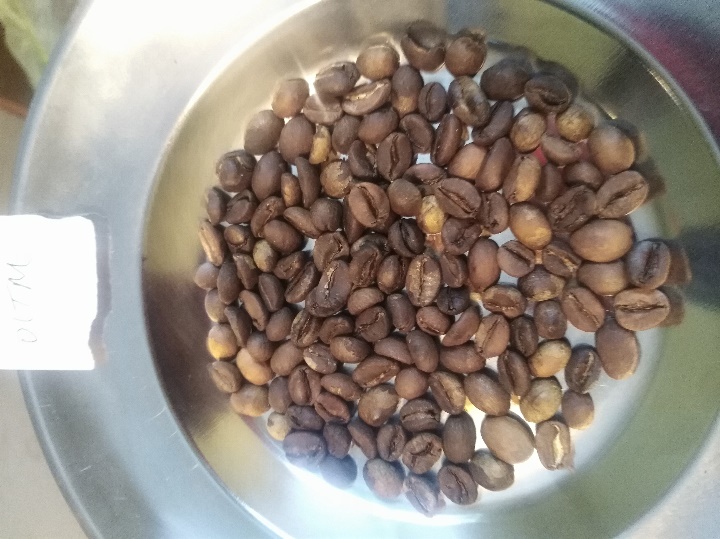

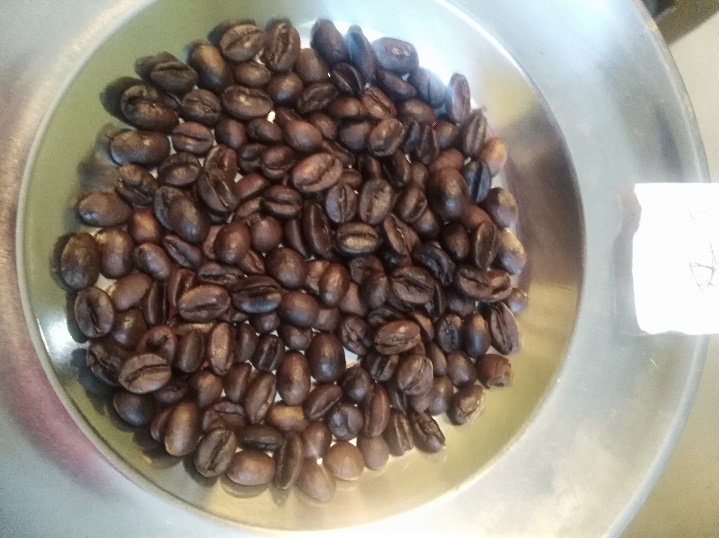


01TD

01TM

01TL
